# A Cross-Linked Cyclosiloxane Polymer Matrix as a Platform Enabling Long-Term Culture of Human Induced Pluripotent Stem Cells with Naïve-Like Features

**DOI:** 10.34133/bmr.0197

**Published:** 2025-04-28

**Authors:** Changjin Seo, Junhyuk Song, Yoonjung Choi, Taemook Kim, Daeyoup Lee, Sangyong Jon

**Affiliations:** ^1^ Department of Biological Sciences, KAIST Institute for the BioCentury, Korea Advanced Institute of Science and Technology (KAIST), Daejeon 34141, Republic of Korea.; ^2^ Center for Precision Bio-Nanomedicine, Korea Advanced Institute of Science and Technology (KAIST), Daejeon 34141, Republic of Korea.; ^3^ Deargen Inc., Daejeon 35220, Republic of Korea.

## Abstract

Culture platforms for human induced pluripotent stem cells (hiPSCs) that rely on feeder cells or extracellular matrices (ECMs) face substantial limitations for practical regenerative medicine applications, including undefined components, high costs, and a tendency to maintain hiPSCs in the primed pluripotent state, which has lower differentiation potential than the naïve state. To overcome these challenges, we developed a long-term hiPSC culture platform based on a cross-linked cyclosiloxane polymer matrix that preserves pluripotency with naïve-like characteristics. Through optimization, we identified an ideal cyclosiloxane polymer matrix, designated as poly-Z, which supported the growth of hiPSCs as spheroids. Even after 60 d of continuous culture, hiPSC spheroids maintained on poly-Z retained pluripotency markers and normal karyotypes at levels comparable to those of hiPSC colonies cultured on conventional vitronectin (VN)-coated plates. Furthermore, mRNA sequencing revealed that hiPSC spheroids cultured on poly-Z not only exhibited up-regulation of typical pluripotency-related genes but also showed increased expression of genes associated with the naïve pluripotent state, in contrast to the primed state observed in hiPSCs cultured on VN-coated plates or in suspension culture. Gene ontology (GO) analysis and gene set enrichment analysis (GSEA) further suggested that the down-regulation of genes involved in cell–ECM interactions contributed to the induction of naïve-like features in poly-Z-cultured hiPSC spheroids. These findings highlight the potential of cross-linked cyclosiloxane-based polymer matrices as an innovative platform for human pluripotent stem cell research and regenerative medicine.

## Introduction

Human induced pluripotent stem cells (hiPSCs) hold great promise in the field of regenerative medicine owing to their pluripotent nature, which enables them to differentiate into various cell types [1]. However, establishing a culture system capable of supporting the long-term proliferation of hiPSCs at a large scale while maintaining their pluripotency is crucial for harnessing the full potential of hiPSCs for practical applications. Among traditional culture systems are suspension cultures of hiPSC spheroids [2–4] and culturing of hiPSCs on mouse feeder cells or extracellular matrix (ECM) Matrigel [5–7]. However, the clinical application of hiPSCs cultured using these latter approaches is limited because of xenogeneic contamination and undefined murine components [8,9]. As an alternative, human recombinant ECM proteins, such as laminin and vitronectin (VN), have been developed for hiPSC culture [7,10,11]. However, their high cost presents challenges for large-scale culture. Accordingly, synthetic polymer matrices have emerged as promising alternatives for hiPSC culture [12–16].

During in vitro culture, hiPSCs exist in 2 pluripotent states: the naïve state and the primed state. Naïve hiPSCs possess greater and broader differentiation potential compared with primed hiPSCs [17]. Moreover, naïve hiPSCs are known to exhibit higher rates of homologous recombination, which facilitates the modifications of specific genes with high accuracy and makes these cells attractive for gene-editing applications [18]. Given these advantageous properties, naïve hiPSCs are highly desirable for various disciplines, including developmental research, regenerative medicine, and disease modeling [18,19]. However, conventional culture methods involving feeder cells, ECM matrices, or suspension culture medium often result in hiPSCs remaining in the primed state. Although several approaches have been developed for converting primed hiPSCs to the naïve state, they often entail complex processes and high costs [20–22], underscoring the need for a simple and cost-effective culture platform that enables the long-term expansion of hiPSCs in the naïve state.

In a recent study, we presented a novel culture platform based on a cyclosiloxane polymer thin film that demonstrated the ability to transform various cancer cell lines into cancer stem cell-like spheroids [23,24]. Through these investigations, we identified the critical role of a specific protein in the cell culture medium and a chemical functionality of the polymer film in driving this transformation. Building upon these findings, we hypothesized that a cyclosiloxane polymer matrix could similarly convert hiPSCs into spheroids while maintaining their pluripotency. However, the cyclosiloxane-based polymer thin film employed in the previous study [23] was created using the initiated chemical vapor deposition method, limiting its scalability and mass production potential. In this study, we devised a new cyclosiloxane polymer matrix that can be facilely prepared using a liquid-based cross-linking method and is highly scalable and suitable for mass production, and evaluated its viability as a platform for the culture of hiPSCs. Using a multifaceted approach, we compared the performance of the newly synthesized polymer matrix to that of the well-established hiPSC culture substrate, VN, or conventional suspension-cultured spheroids in supporting hiPSC growth and pluripotency.

## Materials and Methods

### Preparation of cell culture plates coated with cross-linked cyclosiloxane polymers

Polymer-coated plates with various ratios of cyclosiloxane compounds were prepared through a hydrosilylation reaction with 2,4,6,8-tetramethyl-2,4,6,8-tetravinylcyclotetrasiloxane (V4D4, 99%; Alfa Aesar) and 2,4,6,8-tetramethylcyclotetrasiloxane (TMCTS; Aldrich) in V4D4:TMCTS molar ratios of 4:1, 3:1, 2:1, 1:1, 1:2, and 1:4. TMCTS was treated with platinum(0)-1,3-divinyl-1,1,3,3-tetramethyldisiloxane (Karstedt’s catalyst; Aldrich) at concentrations of 900 parts per million (ppm) (4:1), 200 ppm (3:1), 100 ppm (2:1), 20 ppm (1:1, 1:2), and 10 ppm (1:4). V4D4 was then added at the aforementioned molar ratios and thoroughly mixed. The mixture was dispensed on tissue culture plates at volumes of 10 μl (96-well), 514 μl (6-well), or 5.09 ml (100 mm) and then evenly spread to cover the entire plate bottom. The hydrosilylation reaction was carried out on the plates for 24 h at either 40 °C (1:2, 1:4) or 60 °C (4:1, 3:1, 2:1, 1:1). After the reaction, polymer-coated plates were rinsed 3 times with (in order) isopropyl alcohol, 70% ethanol, and distilled water to remove unreacted substances before being employed for cell culture.

### Surface characterization of cross-linked cyclosiloxane polymer matrices

A Fourier transform infrared (FT-IR) spectrometer (Nicolet iS50; Thermo Fisher Scientific Instrument) was used to measure the FT-IR spectra of V4D4, TMCTS, and cross-linked polymers. Data were obtained using 64 average scans in transmittance mode and recorded in the range of 400 to 4,000 cm^−1^. The chemical composition of the produced polymer was identified using multifunctional x-ray photoelectron spectroscopy (XPS; Sigma Probe, Thermo VG Scientific) at a base pressure of 2.0 × 10^−9^ mbar. Data were recorded in the range of 0 to 1,350 eV using a microfocused monochromator x-ray source (12 kV, KE = 1,486.7 eV). Surface wettability of tissue culture plate, Si wafer, and polymer-coated Si wafer was measured using a contact angle analyzer (SEO Phoenix; Surface Electro Optics) by dropping 10 μl of deionized water on the surface.

### hiPSC culture

The human stem cell lines, CMC-iPSC-003 and CMC-iPSC-009, were provided by the National Stem Cell Bank of Korea (Korea National Institute of Health), originally provided by Catholic University. As a control, hiPSCs were cultured on VN-coated plates (STEMCELL Technologies) in mTeSR plus medium (STEMCELL Technologies). For 3D spheroid culture, hiPSCs were seeded on polymer-coated plates at a density of 5 × 10^4^ cells/cm^2^ and cultured in mTeSR plus medium at 37 °C and 5% CO_2_. For both 2-dimensional (2D)-cultured (VN-coated plates) and 3D-cultured hiPSCs, cells were maintained at 37 °C and 5% CO_2_, and subcultured every 4 d, with changes in medium every 2 d. For subculture of spheroids, hiPSC spheroids were collected in 15-ml conical tubes by centrifuging at 100*g* for 2 min. After the supernatant was drained, ReLeSR dissociation reagent (STEMCELL Technologies) was added to the pellet and incubated at 37 °C for 6 min. The pellets were then collected by centrifuging at 100*g* for 2 min, and the supernatant was removed. Pellets were resuspended in 1 ml of mTeSR plus medium, and hiPSC spheroids were dissociated into cell clumps by filtering through a 40-μm cell strainer. Cell clumps were seeded onto a fresh polymer-coated plate at a density of 5 × 10^4^ cells/cm^2^. Cell proliferation was measured using a hematocytometer after cells were treated with trypan blue solution (Sigma). The proliferation of cells in hiPSC colonies and spheroids was measured after dissociation into single cells using Gentle Cell Dissociation Reagent (STEMCELL Technologies). In a subset of experiments, integrin expression was stimulated by culturing hiPSCs on poly-Z in mTeSR plus medium containing 0.5 mM MnCl_2_ for 24 h. All images of cell morphology were obtained using an inverted microscope (IX53; Olympus). The Institutional Review Board of Korea Advanced Institute of Science and Technology (KAIST-IRB) approved all human pluripotent stem cell experiments (approval number: KH2019-100).

### Suspension culture of hiPSCs using conventional suspension culture medium

For conventional 3D culture, hiPSCs were cultured using StemScale PSC Suspension Medium (Gibco) according to the manufacturer’s instructions. In detail, hiPSCs were seeded on non-tissue culture-treated plates (STEMCELL Technologies) at a density of 1.5 × 10^5^ cells/ml and cultured in StemScale PSC Suspension Medium containing 10 μM Y-27632 (STEMCELL Technologies) at 37 °C and 5% CO_2_. The medium was changed daily for 3 d using a 50% medium exchange method, and cells were subcultured every 4 d. For the subculture of spheroids, hiPSC spheroids were collected in 15-ml conical tubes and collected by centrifuging at 200*g* for 4 min. After the supernatant was aspirated, 1 ml of StemPro Accutase cell dissociation reagent (Gibco) was added to the pellet and incubated at 37 °C for 10 min. Spheroids were dissociated into small clumps by gently triturating pellets 5 to 7 times, after which 3 ml of StemScale PSC Suspension Medium was immediately added. Clumps were pelleted by centrifuging at 200*g* for 4 min, then the supernatant was aspirated, and cell pellets were resuspended in StemScale PSC Suspension Medium containing 10 μM Y-27632. Cell clumps were seeded on non-tissue culture-treated plates at a density of 1.5 × 10^5^ cells/ml, and the above processes were repeated for long-term culture.

### Quantitative real-time polymerase chain reaction

Total RNA was extracted from hiPSCs cultured on VN-coated or polymer-coated plates using Ribospin II (GeneAll), in accordance with the manufacturer’s instructions. Quantitative real-time polymerase chain reaction (qRT-PCR) experiments were carried out on a CFX96 Real-Time PCR Detection System (BIO-RAD) using 100 ng of extracted RNA and LeGene SB-Green One-Step qRT-PCR Master Mix (LeGene Biosciences) in accordance with the manufacturer’s instructions. The list of primers used is provided in Table S1.

### Immunocytochemistry

hiPSCs cultured on VN-coated plates were fixed for 15 min at room temperature in a 4% paraformaldehyde solution (Sigma). Fixed cells were permeabilized for 15 min with 0.2% Triton X-100 (Sigma), then washed with Dulbecco’s phosphate-buffered saline (DPBS), and treated for 30 min at room temperature with 3% bovine serum albumin (BSA) in DPBS. hiPSC spheroids cultured on poly-Z were collected and washed with DPBS and then fixed for 30 min at room temperature in a 4% paraformaldehyde solution. Fixed spheroids were incubated in a 15% sucrose solution and subsequently dehydrated by soaking in a 30% sucrose solution until they sank to the bottom. Following dehydration, frozen blocks were prepared using FSC 22 Clear solution (Leica) and cut into 10-μm-thick slices using a cryotome (CM1850; Leica). Spheroid frozen sections were transferred to microscope slides (Marienfeld), permeabilized for 15 min with 0.2% Triton X-100, then washed with DPBS, and blocked for 30 min at room temperature with 3% BSA in DPBS. All blocked samples were incubated with primary antibody for 12 h at 4 °C, then washed 3 times with DPBS, and incubated with secondary antibody for 1 h at room temperature. The list of antibodies used is provided in Table S2. After again washing 3 times with DPBS, samples were stained with 4′,6-diamidino-2-phenylindole (DAPI) nuclear DNA stain solution (Invitrogen) for 5 min at room temperature. Fluorescence images were obtained with an inverted fluorescence microscope (TI2, Nikon) and a confocal laser scanning microscope (LSM880, Carl Zeiss).

### Flow cytometry

hiPSCs cultured on either N-coated plates or poly-Z-coated plates were dissociated into single cells using the Gentle Cell Dissociation Reagent (STEMCELL Technologies) and then dispersed in fluorescence-activated cell sorting (FACS) buffer [DPBS containing 2% fetal bovine serum (FBS)]. Dissociated cells were immunostained by incubating first with primary antibody for 30 min at 4 °C and then with the appropriate secondary antibody for 30 min at room temperature. The list of antibodies used is provided in Table S2. After incubation, stained cells were collected, washed with DPBS, and redispersed in FACS buffer. The expression of surface markers was assessed using a high-performance multi-parameter flow cytometer (BD LSR Fortessa). Flow cytometry data were analyzed using FlowJo software (Tree Star Inc.).

### Embryoid body formation

For embryoid body (EB) formation, hiPSC spheroids cultured on poly-Z were dissociated into small clumps, seeded into wells of a 6-well Ultra-Low Attachment plate (Corning), and cultured for 7 d using a medium consisting of Dulbecco’s modified Eagle’s medium/nutrient mixture F-12 (DMEM/F-12; Gibco), 20% KnockOut Serum Replacement (SR, Gibco), 1% nonessential amino acid solution (NEAA; Gibco), 2 mm GlutaMAX (Gibco), and 0.1 mM β-mercaptoethanol (Gibco). After EBs had formed, they were transferred to a collagen type 1-coated plate (SPL) and cultured for 7 d in DMEM/F12 medium supplemented with 10% FBS (Gibco). Spontaneous differentiation was confirmed by staining for germline-specific markers for each lineage following the staining procedure outlined in the abovementioned immunocytochemical (ICC) protocol.

### Trilineage differentiation

Trilineage differentiation experiments were carried out using STEMdiff Trilineage Differentiation Kit (STEMCELL Technologies) in accordance with the manufacturer’s instructions. In detail, hiPSC spheroids were dissociated into small clumps and cultured on a Matrigel-coated plate using the corresponding differentiation medium that induces trilineage differentiation. Trilineage differentiation was confirmed by staining for germline-specific markers for each lineage following staining procedure outlined in the abovementioned ICC protocol. For 3D differentiation of hiPSCs on poly-Z, hiPSC were harvested, washed with DPBS, and then cultured on fresh poly-Z using a STEMdiff Trilineage Differentiation Kit. Differentiation was induced according to the manufacturer’s instructions.

### Teratoma assay

For teratoma assays, hiPSC spheroids were dissociated into small clumps, which were then suspended in a 1:1 mixture of DPBS and Matrigel (5 × 10^6^ cells in 100 μl). Small clumps were injected into the right thigh muscle of 8-week-old female nonobese diabetic (NOD)/severe combined immunodeficient (SCID) mice (Orient Bio Inc., Seongnam, South Korea). After 6 weeks, teratomas were excised, sectioned, and stained with hematoxylin and eosin (H&E). Surgical procedures were performed under isoflurane anesthesia to prevent severe suffering. All animal experimental procedures complied with ethical procedures and scientific care and were approved by the Institutional Animal Care and Use Committee of Korea Advanced Institute of Science and Technology (KAIST-IACUC; approval number: KA2023-056-v1).

### Karyotype analysis

After 60 d of culture on poly-Z, hiPSC spheroids were dissociated into small cell clumps and transferred to a VN-coated plate. Samples were then sent to CANCERROP for karyotyping through G-band analysis.

### RNA extraction and mRNA-seq

Total RNA (10 μg) was extracted from hiPSCs cultured on VN- or poly-Z-coated plates using TRIzol Reagent (Invitrogen). Both library construction and mRNA-seq were carried out by Beijing Novogene Bioinformatics Technology Co. Ltd. The libraries were sequenced on an Illumina NovaSeq 6000 platform, and 150-bp paired-end reads were generated. The sequenced reads were aligned to the human reference genome (hg19) using mRNA-seq by expectation maximization (RSEM) software [25]. The rsem-calculate-expression module in RSEM was utilized to quantify the count of aligned reads and transcript per million (TPM) genes. Differentially expressed genes (DEGs; adjusted *P* value < 0.05 and log_2_ fold change > |1|) were investigated using the R package, DESeq2, and visualized as heatmaps and volcano plots.

### Gene ontology analysis and gene set enrichment analysis

Gene ontology (GO) analysis of DEGs was performed using the Enrich online web tool [26]. Kyoto Encyclopedia of Genes and Genomes (KEGG) pathway analysis was performed using GSEA software (version 4.2.3) with default values for other parameters [27]. Gene expression levels and DEGs were visualized as heatmaps using Morpheus (https://software.broadinstitute.org/morpheus/) and the R package, pheatmap. Bar plots were created using the R package, ggplot2.

### Western blot analysis

Total protein was extracted from hiPSCs cultured on VN-coated plates, in suspension, or on poly-Z-coated plates using PRO-PREP protein extraction solution containing a protease inhibitor cocktail (iNtRON Biotechnology). Cells were lysed on ice for 20 min. Protein concentrations in the lysates were quantified using the BCA Protein Assay Kit (Thermo Fisher Scientific). Equal amounts of protein (20 μg) were separated by electrophoresis using the Mini-PROTEAN Tetra System (Bio-Rad) and subsequently transferred onto polyvinylidene difluoride (PVDF) membranes using the Tetra Blotting Module (Bio-Rad), following the manufacturer’s instructions. Membranes were blocked with 3% BSA blocking solution for 1 h at room temperature and then incubated with primary antibodies at 4 °C for 12 h. After 3 washes with tris-buffered saline with Tween 20 (TBST), membranes were incubated with the appropriate secondary antibody for 1 h at room temperature. The list of antibodies used is provided in Table S2. Following incubation, membranes were washed 5 times with TBST, and protein bands were detected using Amersham ECL Prime Western Blotting Detection Reagents (Cytiva) and visualized with a ChemiDoc MP system (Bio-Rad).

### Statistical analysis

All data were expressed as means ± SD. Student’s *t* test (for 2 groups) and a one-way analysis of variance (ANOVA) (for 3 or more groups) with Tukey’s multiple comparisons test were used for statistical analyses. Analyses were carried out using GraphPad Prism v.8.2. A *P* value of <0.05 was considered statistically significant.

## Results

### Construction and characterizations of cross-linked cyclosiloxane polymer-coated plates

**Fig. 1.**
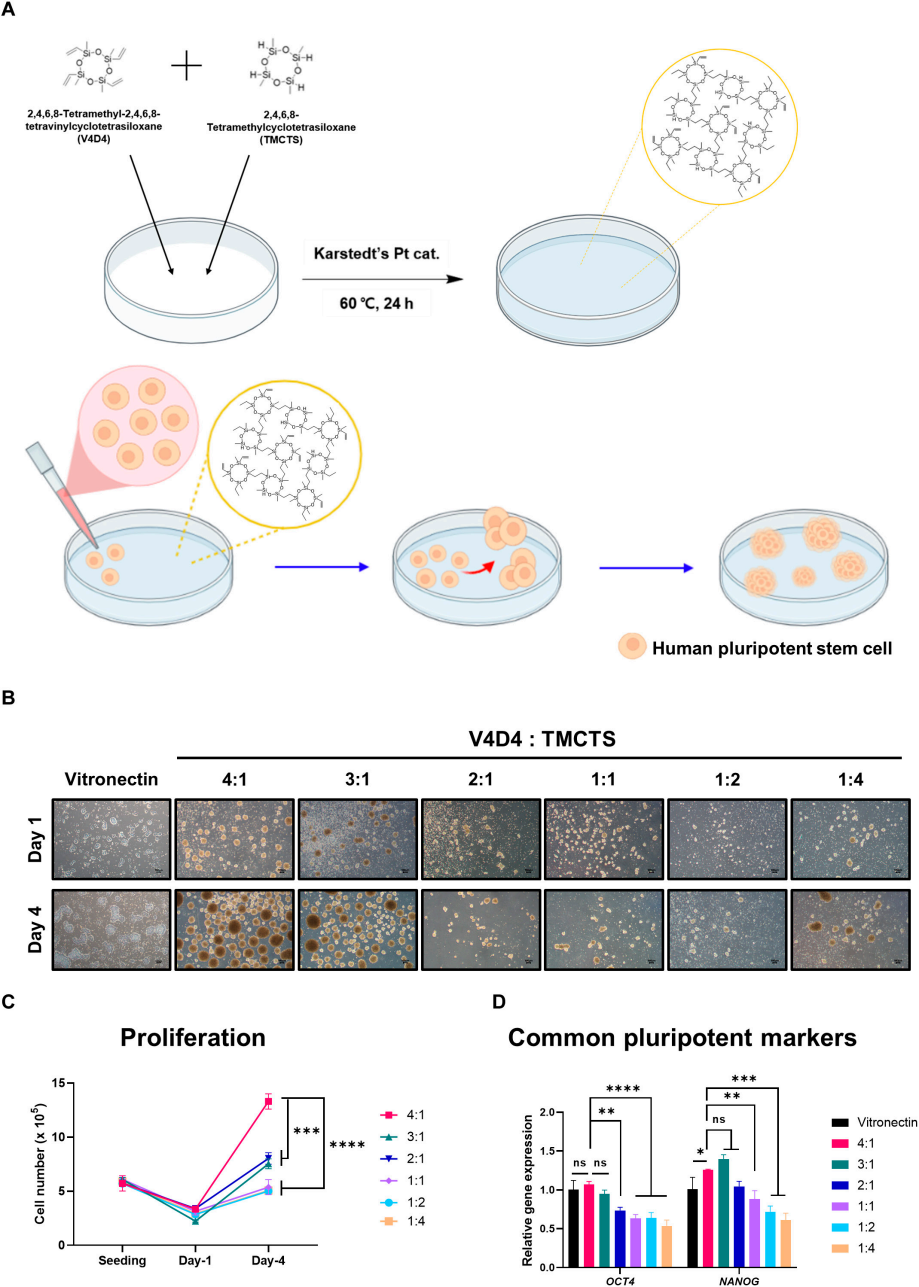
Construction and screening of cyclosiloxane-based polymer matrices for hiPSC culture. (A) Schematic illustration of the synthesis of a cross-linked cyclosiloxane polymer matrix-coated plate and the formation of hiPSC (CMC-iPSC-009) spheroids on the plate. (B to D) Morphology (B), cell proliferation (C), and expression ratios of pluripotency genes (D) of hiPSCs (CMC-iPSC-009) cultured for 1 and 4 d on VN-coated and 6 different cross-linked cyclosiloxane polymer-coated plates (5 × 10^4^ cells/cm^2^). Scale bar, 100 μm. All experiments were conducted in triplicate. The results in (C) and (D) represent the means ± SD of 3 independent experiments (ns: not significant; *P < 0.05, **P < 0.01, ***P < 0.001, ****P < 0.0001).

We previously developed a cell culture plate in which a cyclosiloxane polymer thin film coating was applied using the initiated chemical vapor deposition method [23]. However, polymer-coated plates are difficult to mass produce with this method. As an alternative, we developed an approach for coating cell culture plates with an analogous cyclosiloxane polymer in the liquid phase. In this simpler approach for preparing polymer matrix-coated plates, we cross-linked 2 cyclosiloxane monomers—V4D4 and TMCTS—on a tissue culture plate through a hydrosilylation reaction at 40 to 60 °C using Karstedt’s catalyst (Fig. 1A) [28]. Both V4D4 and TMCTS have 4 functional groups for hydrosilylation; thus, polymer matrices were fabricated by controlling the ratio of the 2 monomers. Specifically, a series of cross-linked cyclosiloxane polymer matrices was fabricated by varying the ratio of V4D4 and TMCTS from 4:1 to 1:4 and then characterized using water contact angle, FT-IR, and XPS. All as-prepared cyclosiloxane polymer matrices exhibited substantially increased water contact angles (>37°) compared with a conventional tissue culture plate (Fig. S1), indicating an increase in hydrophobicity. FT-IR analyses confirmed that the polymer matrix was formed by a hydrosilylation reaction catalyzed by Pt, as indicated by a marked decrease in the characteristic peaks of the vinyl functional group in the 1,400 to 1,600 cm^−1^ region (Fig. S2). In addition, XPS analysis revealed that, as the ratio of V4D4 in polymer matrices decreased, the ratio of carbon atoms decreased, while the ratios of oxygen and silicon atoms increased (Fig. S3 and Table S3).

### Determining the optimal polymer matrix for hiPSC culture

To establish the optimal composition of the 2 monomers in the polymer matrices, we cultured hiPSCs on cross-linked cyclosiloxane polymer-coated plates with 6 different V4D4/TMCTS ratios and assessed their efficacy in terms of cell morphology, proliferation ability, and pluripotency-related gene expression levels compared with the conventional VN-coated plate hiPSC 2D culture system. On conventional VN-coated plates, hiPSCs grew into colonies after 4 d of culture; in contrast, hiPSCs cultured on cross-linked cyclosiloxane polymer matrices formed spheroids regardless of the composition of the 2 monomers (Fig. 1B). Among the 6 polymer-coated plates, the polymer matrix derived from a V4D4/TMCTS ratio of 4:1 supported the greatest spheroid-forming ability. Furthermore, hiPSCs grown on plates with this 4:1 ratio exhibited the highest rate of cell proliferation (Fig. 1C) and the highest expression of the pluripotency-associated genes, *OCT4* and *NANOG*, on day 4 (Fig. 1D). Notably, another hiPSC line also demonstrated the ability to grow as spheroids on a polymer matrix derived from a 4:1 ratio while retaining pluripotency (Fig. S4). Although the 3:1 ratio-derived polymer matrix performed as well as the 4:1 ratio in terms of spheroid-forming ability and gene expression, taking all 3 categories of evaluation together, the 4:1 ratio-derived cross-linked cyclosiloxane polymer, which we named “poly-Z”, was found to be the most suitable matrix for hiPSC culture.

### hiPSCs maintain pluripotency during long-term culture on poly-Z

**Fig. 2.**
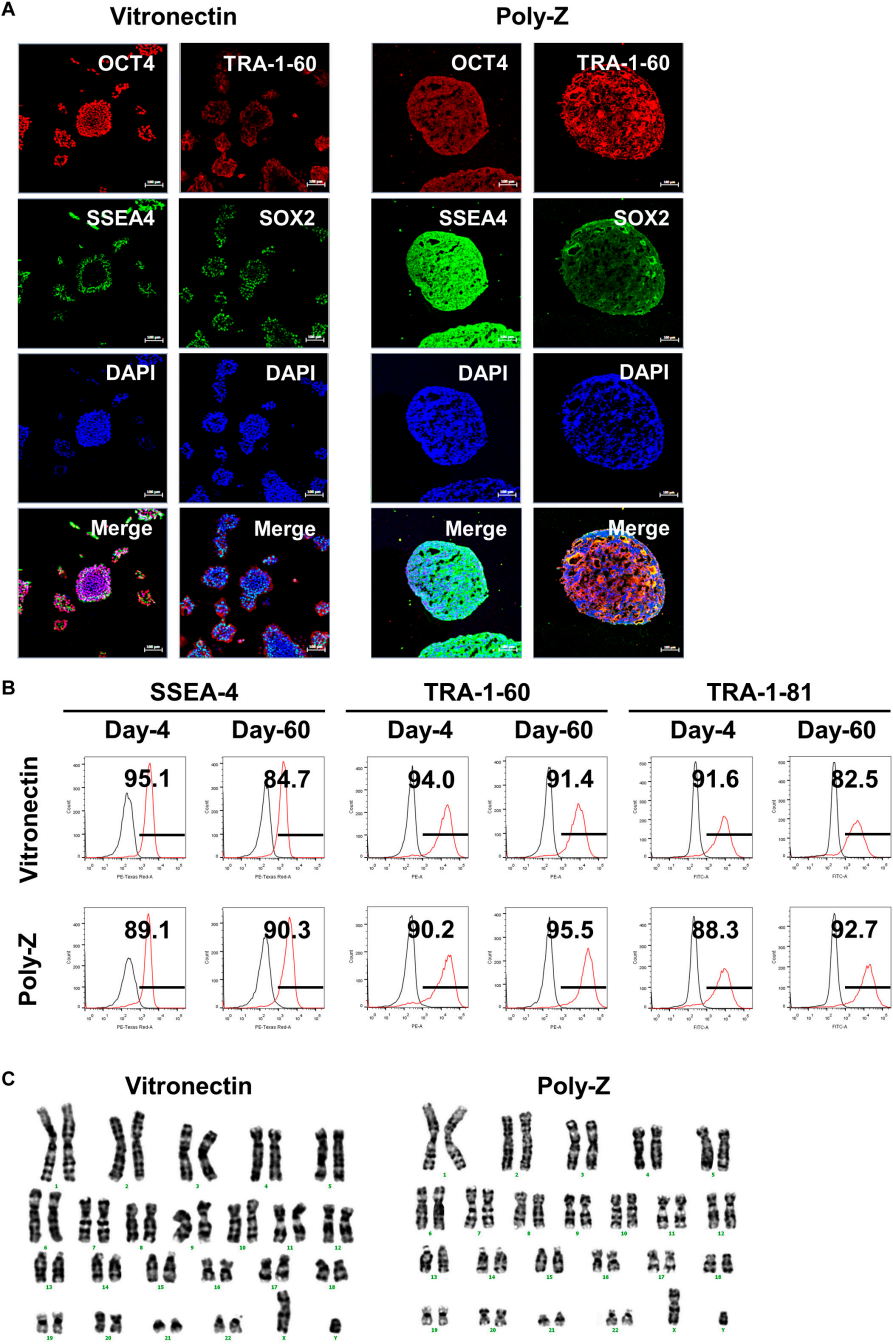
Pluripotency of hiPSC spheroids cultured long-term on poly-Z. (A) ICC staining of representative pluripotency markers in hiPSCs (CMC-iPSC-009) cultured for 60 d on VN or poly-Z. Scale bar, 100 μm. (B) Flow cytometry analysis of SSEA-4, TRA-1-60, and TRA-1-81 expression in hiPSCs (CMC-iPSC-009) cultured on VN or poly-Z for 4 and 60 d. (C) Karyotype analysis of hiPSCs (CMC-iPSC-009) after 60 d of culture on VN or poly-Z. All experiments were performed in triplicate.

To determine whether poly-Z-cultured hiPSC spheroids retain pluripotency during long-term culture, we assessed the expression of pluripotency-related proteins after 60 d of culture on poly-Z- or VN-coated plates. ICC staining of hiPSC colonies on VN and hiPSC spheroids on poly-Z revealed robust expression of the pluripotency markers OCT4, SSEA-4, TRA-1-60, and SOX2 in both conditions (Fig. 2A). Additionally, flow cytometry analysis confirmed that the proportion of cells expressing the pluripotency surface markers SSEA-4, TRA-1-60, and TRA-1-81 remained stable in both VN-cultured hiPSC colonies and poly-Z-cultured hiPSC spheroids over long-term culture (Fig. 2B). Notably, after 60 d, poly-Z-cultured hiPSC spheroids exhibited a higher proportion of cells expressing SSEA-4 and TRA-1-81 compared to VN-cultured hiPSC colonies. Given that chromosomal abnormalities can arise during extended hiPSC culture, we next examined the karyotype of long-term cultured hiPSC spheroids on poly-Z. As shown in Fig. 2C, poly-Z-cultured hiPSC spheroids maintained a normal karyotype, indicating the absence of genetic abnormalities or chromosomal changes even after prolonged culture. These findings demonstrate that poly-Z serves as an effective polymer matrix for sustaining hiPSC pluripotency during long-term culture.

### Long-term cultured hiPSC spheroids on poly-Z differentiate into 3 germ layers

**Fig. 3.**
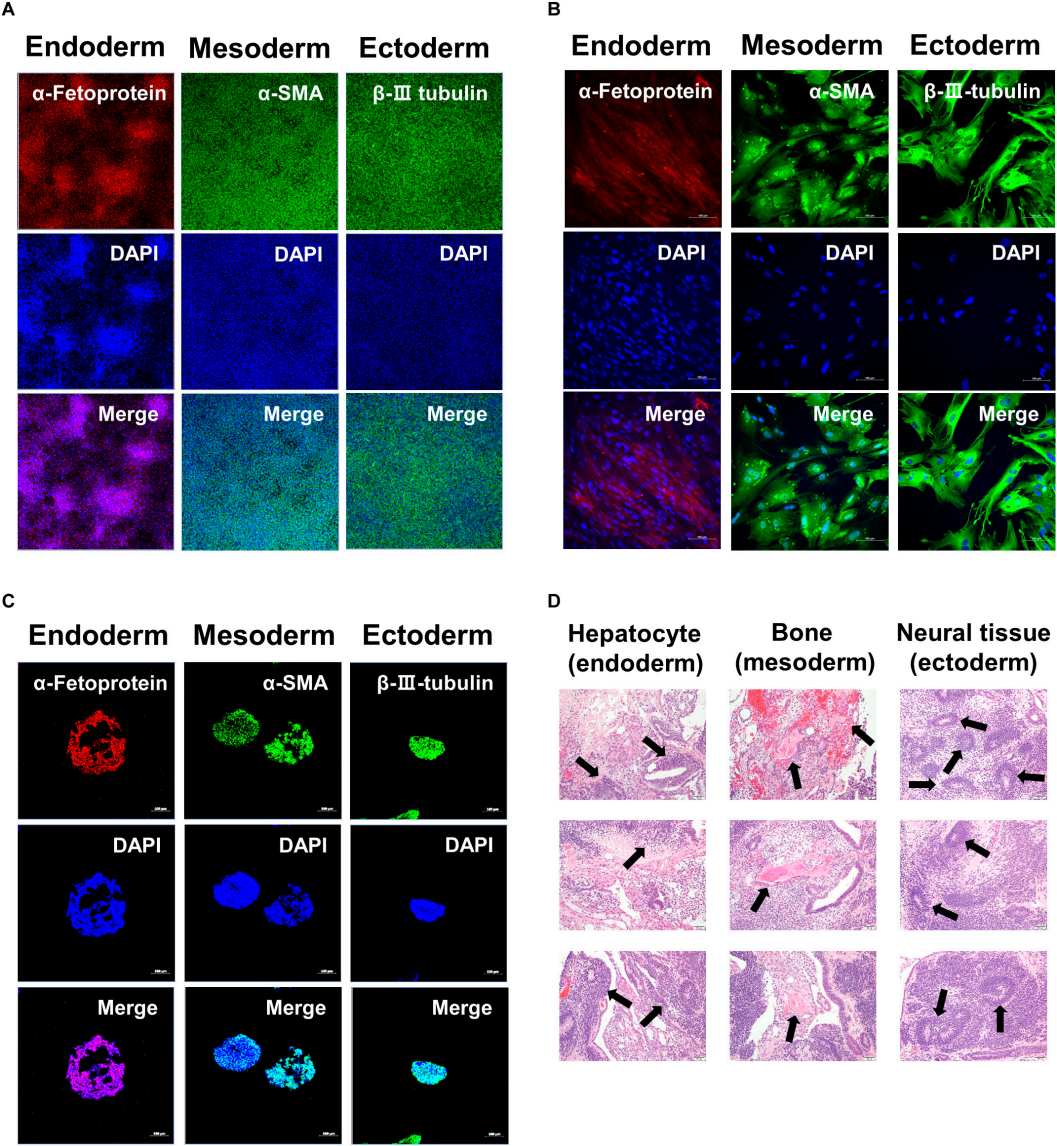
Capacity of poly-Z-cultured hiPSC spheroids to differentiate into 3 germlines. ICC detection of 3 germ layer markers following trilineage differentiation of (A) dissociated hiPSC (CMC-iPSC-009) spheroids on Matrigel-coated tissue culture plates, (B) embryoid bodies constructed from dissociated hiPSC (CMC-iPSC-009) spheroids, and (C) hiPSCs (CMC-iPSC-009) spheroids on poly-Z. (D) Staining of teratomas with hematoxylin and eosin (H&E) after injection of dissociated hiPSC (CMC-iPSC-009) spheroids. Marker proteins for each germ layer: α-fetoprotein (endoderm), α-SMA (mesoderm), and β-III-tubulin (ectoderm). All experiments were performed in triplicate. Scale bars, 100 μm (A to C) and 50 μm (D).

To evaluate the differentiation capacity of hiPSC spheroids cultured on poly-Z, we conducted 3 functional differentiation assays: in vitro-directed differentiation into 3 germ layers, EB formation assays, and in vivo teratoma assays. For in vitro-directed differentiation, hiPSC spheroids cultured for 60 d were dissociated into single cells, which were then cultured on a Matrigel-coated plate using medium that induces differentiation into each of the 3 germ layers. As depicted in Fig. 3A, poly-Z-cultured hiPSCs successfully differentiated into the 3 germ layers—endoderm, mesoderm, and ectoderm—demonstrating differentiation efficiency comparable to that of VN-cultured hiPSCs (Fig. S5). Using an EB formation assay, we confirmed the capacity for spontaneous differentiation of poly-Z-cultured hiPSC spheroids into the 3 germ layers (Fig. 3B). Next, we examined whether the spheroid form of hiPSCs on poly-Z can directly differentiate into 3 germ layers without first being dissociated into single cells. Treatment of hiPSC spheroids on poly-Z with media that induce differentiation into each of the 3 germ layers resulted in high-efficiency, direct differentiation of spheroids into the corresponding 3 germ layers (Fig. 3C), indicating the utility of hiPSC spheroids for practical applications in regenerative medicine. In the in vivo teratoma assay, poly-Z-cultured hiPSC spheroids were capable of differentiating into hepatocytes in the endoderm, neural tissue in the ectoderm, and bone in the mesoderm (Fig. 3D), exhibiting a differentiation capacity similar to that of VN-cultured hiPSCs (Fig. S6). These findings suggest that hiPSC spheroids cultured on poly-Z maintain pluripotency and can differentiate into 3 germ layers, even after long-term culture.

### The gene expression profile of poly-Z-cultured hiPSC spheroids indicates a naïve-like state

**Fig. 4.**
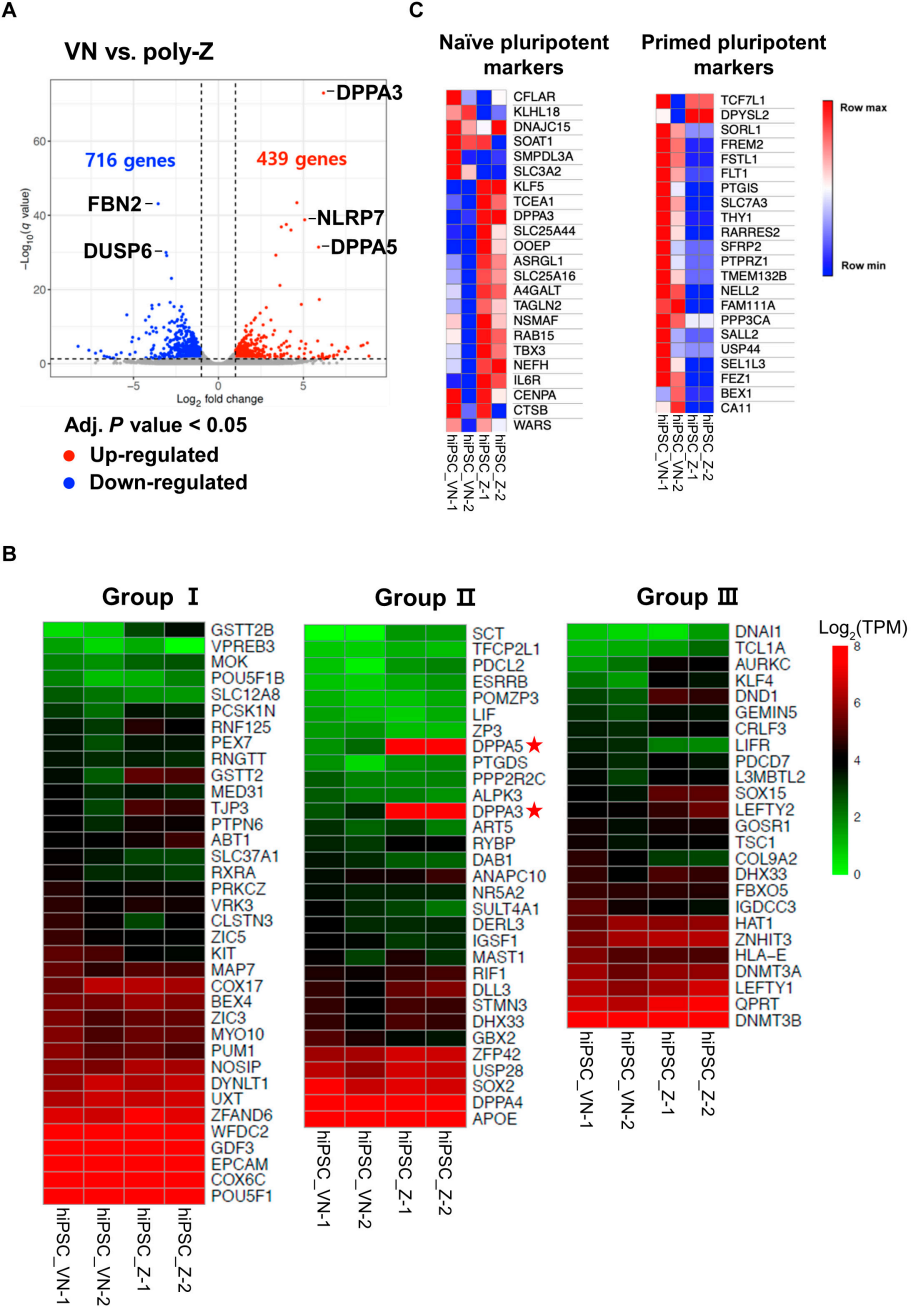
Comparison of gene expression profiles using mRNA-seq. (A) Differences in gene expression between poly-Z- and VN-cultured hiPSCs (CMC-iPSC-009), presented as a volcano plot. Two biological replicates were analyzed. DEGs exhibiting fold changes greater than 2 are indicated in red (up-regulated) and blue (down-regulated). The up-regulated genes, indicated by red dots, are associated with naïve pluripotency, while the down-regulated genes, represented by blue dots, are linked to primed pluripotency. (B) Heatmap of gene expression in poly-Z- and VN-cultured hiPSCs (CMC-iPSC-009). Group I genes are up-regulated in embryonic stem cells (ESCs) and iPSCs. Genes in group II are up-regulated more in ESCs and iPSCs than in partially induced PSCs. Genes in group III are up-regulated more in ESCs than in iPSCs. (C) Comparison of expression of naïve- and primed-state pluripotency markers between poly-Z- and VN-cultured hiPSCs (CMC-iPSC-009).

Using high-throughput mRNA-sequencing (mRNA-seq), we compared the gene expression profile of hiPSC spheroids cultured on poly-Z for 60 d with that of hiPSCs cultured on VN-coated plates. mRNA-seq data revealed 439 up-regulated and 716 down-regulated genes in the poly-Z group compared with VN-cultured hiPSCs (*P* < 0.5; Fig. 4A). For gene sets known to be up-regulated in hiPSCs [1,29], the poly-Z group showed expression of typical pluripotent stem cell marker genes at levels similar to those of the VN group (Fig. 4B). Interestingly, *DPPA3* and *DPPA5*, which have been reported as markers for the naïve hiPSCs state [30–33], were substantially up-regulated in the poly-Z group compared with the VN group (Fig. 4B, middle heatmap). To determine whether poly-Z-cultured hiPSCs enhanced the features of naïve pluripotency, we analyzed the expression of gene sets associated with the naïve or primed states of hiPSCs [34]. Whereas VN-cultured hiPSCs exhibited up-regulation of genes related to the primed state of pluripotency, poly-Z-cultured hiPSCs exhibited up-regulation of genes related to the naïve state of pluripotency (Fig. 4C).

### Comparison of naïve state-associated gene expression between poly-Z-cultured hiPSC spheroids and hiPSCs cultured using conventional methods

**Fig. 5.**
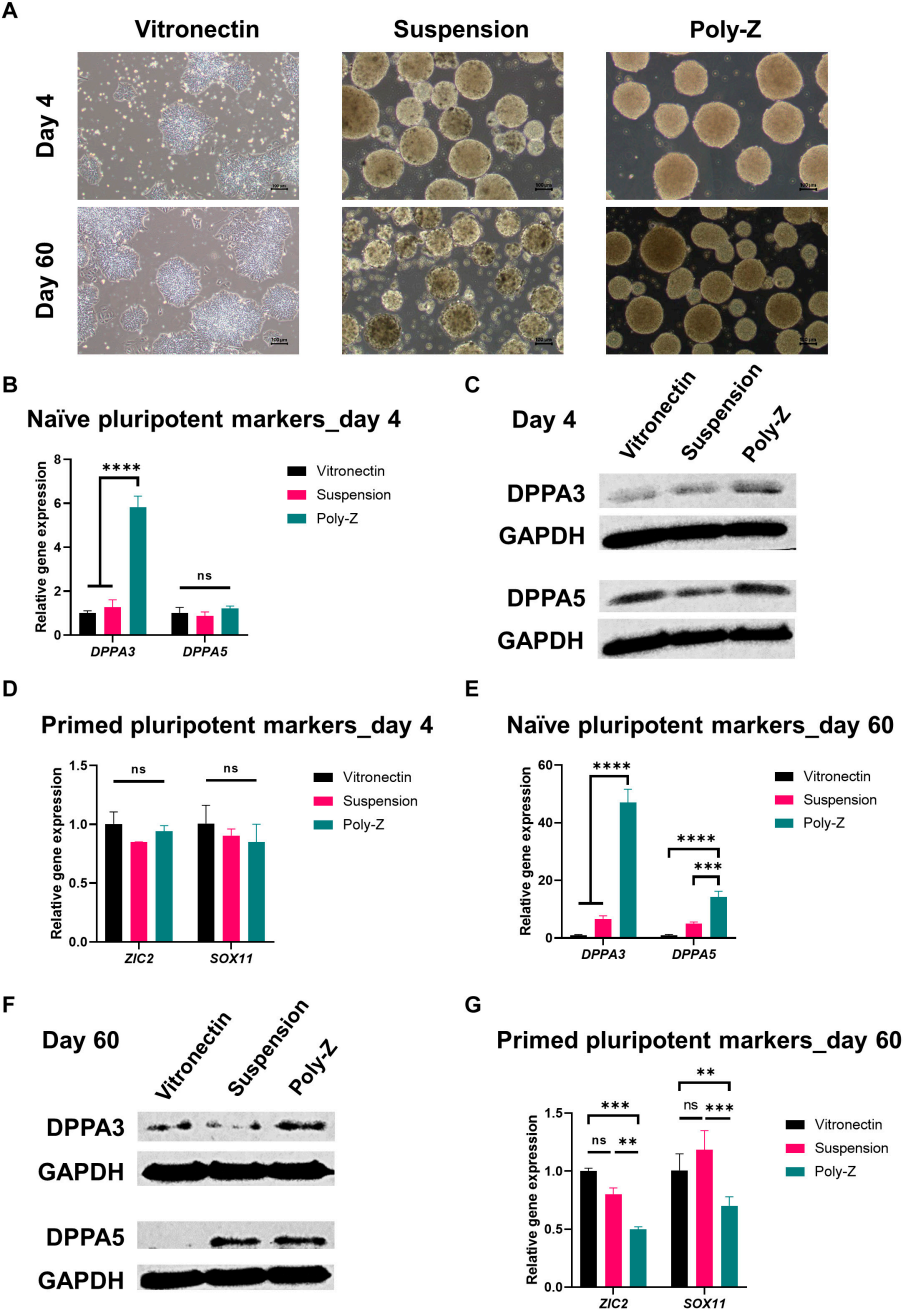
Evaluation of naïve and primed pluripotency marker expression in VN-, suspension-, and poly-Z-cultured hiPSCs. (A) Cell morphologies of hiPSC (CMC-iPSC-009) spheroids cultured for 4 or 60 d on poly-Z or in suspension culture. Scale bar, 100 μm. (B) Relative expression of naïve pluripotency genes in hiPSCs (CMC-iPSC-009) cultured on VN, suspension culture, or poly-Z for 4 d. (C) Western blot analysis of naïve pluripotency markers in hiPSCs (CMC-iPSC-009) cultured on VN, suspension culture, or on poly-Z for 4 d. (D) Relative expression of primed pluripotency genes in hiPSCs (CMC-iPSC-009) cultured on VN, suspension culture, or poly-Z for 4 d. (E) Relative expression of naïve pluripotency genes in hiPSCs (CMC-iPSC-009) cultured on VN, in suspension, or on poly-Z for 60 d. (F) Western blot analysis of naïve pluripotency markers in hiPSCs (CMC-iPSC-009) cultured on VN, in suspension, or on poly-Z for 60 d. (G) Relative expression of primed pluripotency genes in hiPSCs (CMC-iPSC-009) cultured on VN, in suspension, or on poly-Z for 60 d. All experiments were performed in triplicate. The results in (B) to (E) represent the means ± SD of 3 independent experiments (ns: not significant; **P < 0.01, ***P < 0.001, ****P < 0.0001).

Because hiPSCs can be cultured as spheroids using a conventional suspension culture medium [2–4], we also compared the features of poly-Z-cultured hiPSC spheroids with conventional suspension-cultured hiPSC spheroids and VN-cultured hiPSC colonies after short-term (day 4) and long-term (day 60) culture. Although little differences were observed in the formation rate or size of hiPSC spheroids between poly-Z and the suspension culture method, there was a distinct difference in the morphology of spheroids (Fig. 5A and Fig. S7). Whereas uniform, compact spheroids were formed on poly-Z, unidentified black spots inside the spheroids and dead cells were frequently observed in the hiPSC spheroids formed in suspension culture after 60 d of culture. We next compared the cell proliferation capacity of hiPSC spheroids cultured on poly-Z or in suspension at each passage, performed at 4-day intervals. hiPSC spheroids cultured on poly-Z showed an increased proliferation rate compared to that of hiPSC spheroids cultured in suspension (Fig. S8 and Table S4). Moreover, the cell proliferation rate was maintained in the poly-Z system even in late passages, whereas this rate gradually decreased in suspension culture. Collectively, these results indicate the superior proliferation capacity of hiPSCs cultured using the poly-Z method compared with conventional suspension culture. We then compared the expression of common pluripotency marker genes on day 60 of long-term culture. Both hiPSC spheroids cultured on poly-Z and in suspension showed expression of pluripotency marker genes similar to that of VN-cultured hiPSCs (Fig. S9), confirming maintenance of pluripotency, even after long-term culture.

Next, we compared the expression of naïve state-associated gene sets in poly-Z-cultured hiPSC spheroids with those in 2 conventional hiPSC culture methods at days 4 and 60. On day 4, among the naïve pluripotency markers, *DPPA3* exhibited a substantial increase (~6-fold) in expression in poly-Z-cultured hiPSC spheroids compared to VN-cultured hiPSCs or suspension-cultured hiPSC spheroids. In contrast, *DPPA5* expression showed no significant difference among the 3 groups (Fig. 5B). At the protein level, DPPA3 was most highly expressed in poly-Z-cultured hiPSC spheroids, whereas DPPA5 levels remained comparable across all culture conditions (Fig. 5C). Furthermore, on day 4, there were minimal differences in the expression levels of primed pluripotency markers among the 3 culture methods (Fig. 5D). However, after 60 d of long-term culture, the expression patterns of naïve and primed pluripotency markers diverged substantially in poly-Z-cultured spheroids compared to the conventional hiPSC culture methods. Both *DPPA3* and *DPPA5*, key naïve state-associated genes, were markedly up-regulated in poly-Z-cultured hiPSC spheroids compared to VN-cultured hiPSCs and suspension-cultured hiPSC spheroids (Fig. 5E). At the protein level, both DPPA3 and DPPA5 exhibited the highest expression in poly-Z-cultured hiPSC spheroids relative to the other 2 culture conditions (Fig. 5F). In contrast, expression of the primed pluripotency marker genes, *ZIC2* and *SOX11*, was significantly down-regulated in poly-Z-cultured hiPSC spheroids compared with the 2 conventional hiPSC culture methods (Fig. 5G). Taken together, these findings suggest that poly-Z can maintain the pluripotency and promote the naïve-like features of hiPSCs, unlike other conventional hiPSC culture methods, which remain hiPSCs in the primed state.

### Induction of a naïve-like features in poly-Z-cultured hiPSC spheroids is associated with the down-regulation of ECM organization

**Fig. 6.**
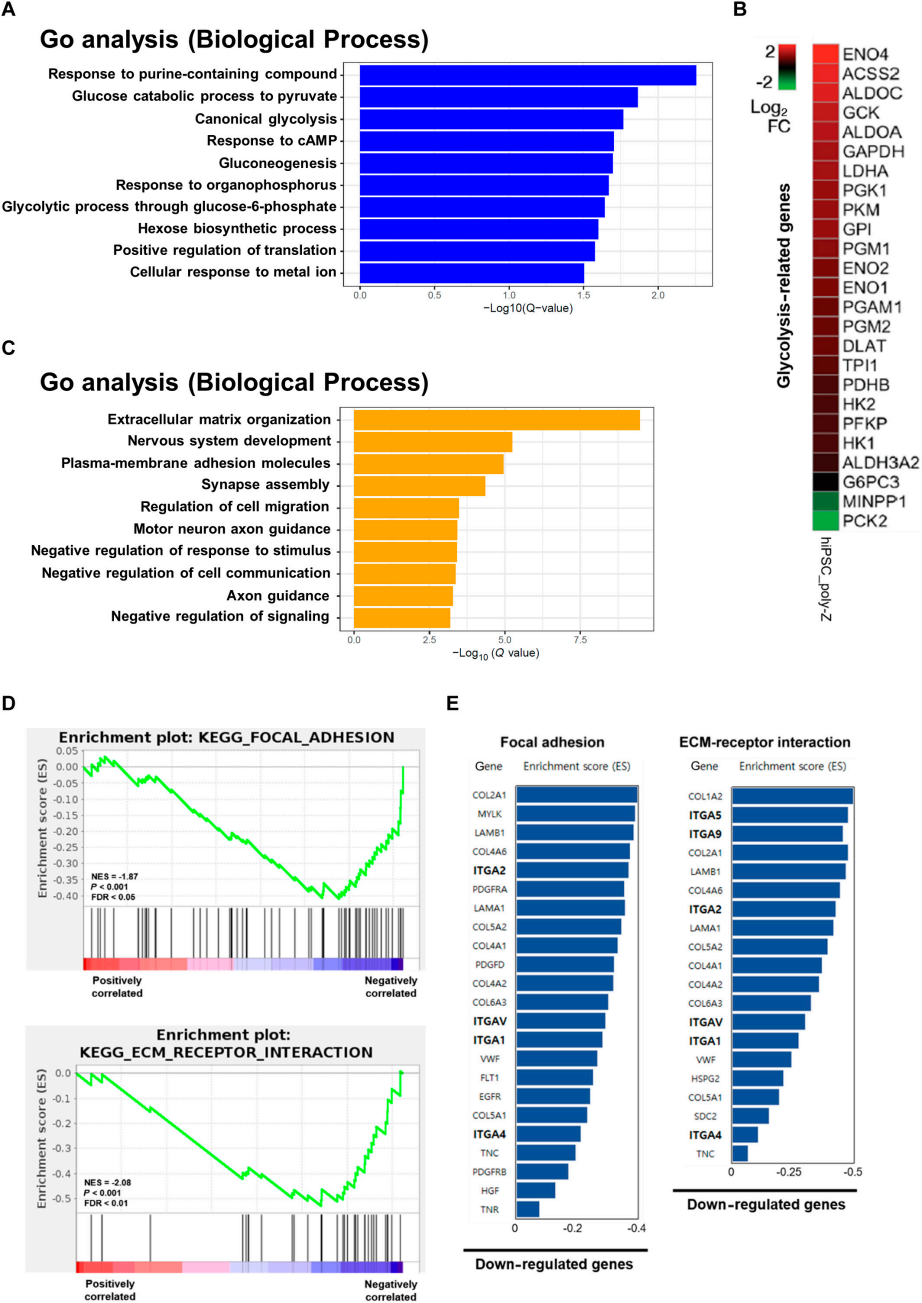
Analysis of GO and gene set enrichment in poly-Z-cultured hiPSC spheroids. (A) GO analysis of biological processes up-regulated in poly-Z-cultured hiPSC (CMC-iPSC-009) spheroids. (B) Heatmap displaying glycolysis-related genes in poly-Z-cultured hiPSC (CMC-iPSC-009) spheroids. Expression levels are presented as relative (log_2_) values normalized to control signals. (C) GO analysis of biological processes down-regulated in poly-Z-cultured hiPSC (CMC-iPSC-009) spheroids. (D) Gene set enrichment analysis (GSEA) of the transcriptome in poly-Z-cultured hiPSC (CMC-iPSC-009) spheroids versus VN-cultured hiPSC (CMC-iPSC-009) spheroids. (E) List of genes with negative correlations between focal adhesion and ECM–receptor interaction terms identified using GSEA.

To investigate the primary biological processes associated with the induction of a naïve-like features in poly-Z-cultured hiPSC spheroids, we performed a GO analysis. Intriguingly, we discovered that most glycolysis-related biological processes were up-regulated (adjusted *P* value = 0.05) in poly-Z-cultured hiPSCs (Fig. 6A). In fact, heatmap data revealed that the glycolysis-related gene set was notably up-regulated in poly-Z-cultured hiPSCs (Fig. 6B). This finding is consistent with previous reports that glycolysis is the primary metabolic pathway involved in naïve state pluripotency and cell reprogramming [35,36]. In addition, we discovered that ECM organization processes were substantially down-regulated in poly-Z-cultured hiPSCs (Fig. 6C). Moreover, a gene set enrichment analysis (GSEA) revealed a marked decrease in the expression of genes related to cell–ECM interactions, such as focal adhesion and ECM–receptor interactions (Fig. 6D). A reduction in the expression of genes encoding several integrin subtypes and ECMs (Fig. 6E and Fig. S10) provided further confirmation.

**Fig. 7.**
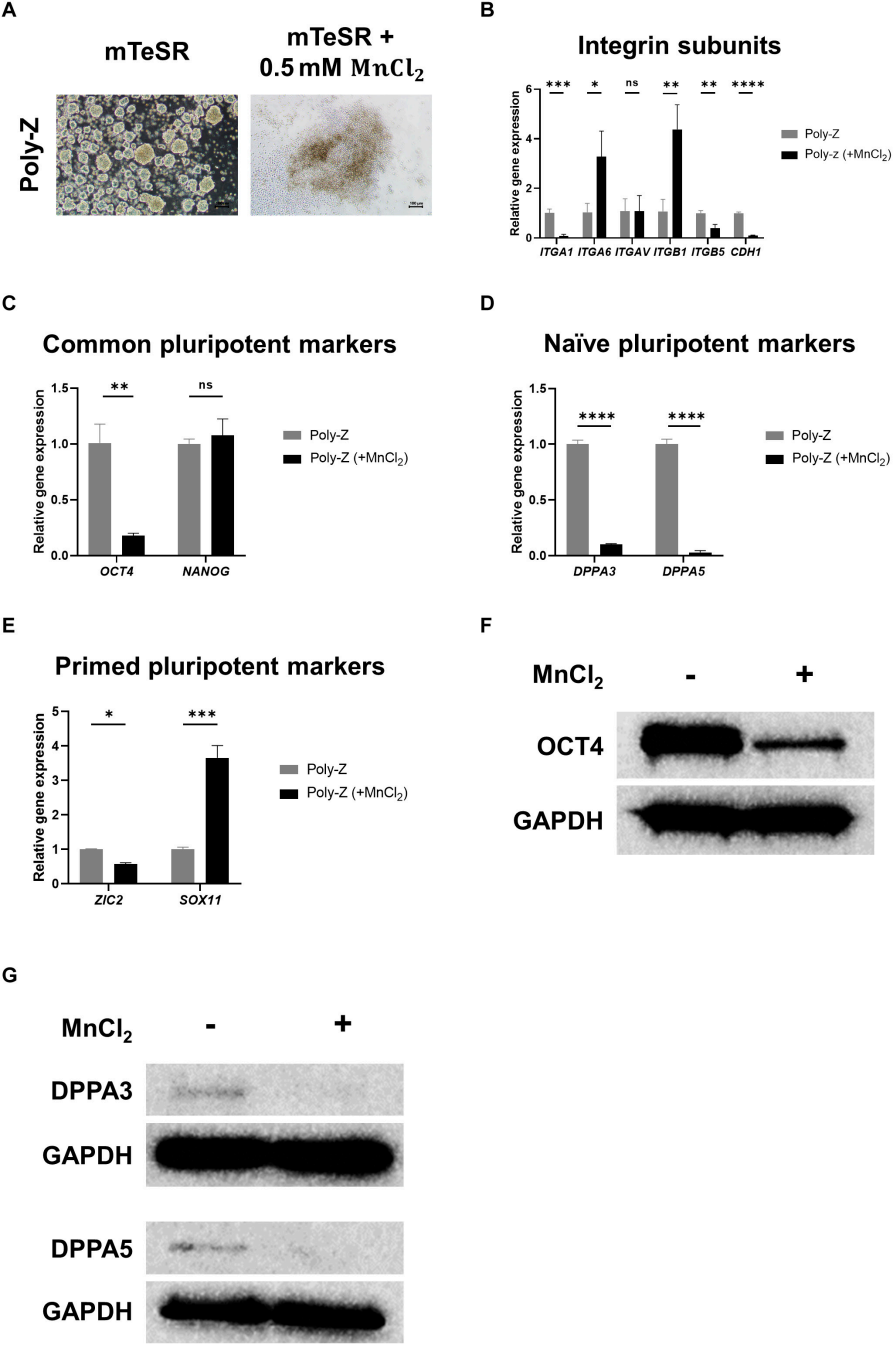
Effects of MnCl_2_ treatment, to activate integrin expression, on spheroid formation and gene expression in hiPSCs cultured on poly-Z. (A to E) Morphology (A) and relative expression of integrin subunit genes (B), self-renewal genes (C), naïve pluripotency genes (D), and primed pluripotency genes (E) in hiPSCs (CMC-iPSC-009) cultured on poly-Z, with and without MnCl_2_ treatment. (F and G) Western blot analysis of a common pluripotency marker (F) and naïve pluripotency markers (G) in hiPSCs (CMC-iPSC-009) cultured on poly-Z, with or without MnCl_2_ treatment. Scale bar, 100 μm. All experiments were performed in triplicate. Results from the 3 independent experiments in (B) to (E) are presented as means ± SD (ns: not significant; *P < 0.05, **P < 0.01, ***P < 0.001, ****P < 0.0001).

On the basis of these findings, we speculated that the increased naïve-like features in poly-Z-cultured hiPSC spheroids was attributable to the decreased interaction between ECM and integrin. According to previous research, a decrease in focal adhesion promotes ground pluripotency in mouse embryonic stem cells and a decrease in cell-matrix adhesion improves pluripotency in hiPSCs [37–39]. To investigate the effect of ECM–integrin interactions on the promotion of naïve-like features by poly-Z culture, we cultured hiPSCs on poly-Z for 24 h in the presence of manganese (Mn^2+^) ions (added to the culture medium as MnCl_2_), reflecting the fact that Mn^2+^ is known to potently promote integrin expression [40]. Surprisingly, hiPSC spheroids did not form on poly-Z when Mn^2+^ was present (Fig. 7A). With Mn^2+^ supplementation, the expression of *ITGA6* and *ITGB1* genes increased substantially, whereas expression of the *CDH1* gene, which is involved in cell-to-cell interactions, decreased significantly (Fig. 7B). Moreover, we found that expression of the gene for the common pluripotency marker, *OCT4*, and 2 naïve pluripotency markers, *DPPA3* and *DPPA5*, was dramatically decreased in the presence of Mn^2+^, whereas the primed pluripotency marker, *SOX11*, was significantly up-regulated (Fig. 7C to E). At the protein level, the expression of OCT4, DPPA3, and DPPA5 was remarkably reduced in the presence of Mn^2+^, aligning with the gene expression results (Fig. 7F and G). These results suggest that down-regulated interactions between integrins on cells and ECMs in poly-Z-cultured hiPSC spheroids play a crucial role in the promotion of naïve-like pluripotency.

## Discussion

Despite the development of various culture platforms for hiPSCs, practical applications still face certain limitations that need to be overcome [41,42]. One prominent constraint associated with conventional hiPSC culture platforms is the predominance of a primed pluripotent state in the resulting hiPSCs [21,43,44]. Although it is possible to convert these primed-state hiPSCs into a naïve state through transgene insertion or treatment with cytokines or inhibitors [20–22], these approaches are complex and costly. In this study, we have introduced a simple culture platform based on a cross-linked cyclosiloxane polymer matrix that can be prepared in a liquid phase capable of mass production. This platform enables long-term culture of hiPSCs in a spheroid form while promoting the naïve-like features—all without the need for additional reagents or gene insertions. Within our experimental settings, the poly-Z material exhibited superior performance in all aspects of hiPSC evaluation compared with conventional VN-coated plates or spheroid culture in suspension.

One intriguing aspect of our polymer matrix, poly-Z, is its ability to support the culture of hiPSCs in spheroids, distinguishing it from conventional adherent colony-forming culture methods. While the morphology of hiPSCs cultured on poly-Z may differ from that of VN-cultured hiPSC colonies, they maintain expression of pluripotency proteins and retain the capacity to differentiate into all 3 germ cell types (Figs. 2 and 3). Notably, hiPSC spheroids cultured on poly-Z can undergo direct differentiation into the 3 germ cell lineages without the need for dissociation into single cells (Fig. 3C), which is advantageous for practical applications. Although both poly-Z and conventional suspension culture of hiPSCs as spheroids maintained the expression of pluripotency genes during long-term culture, the cell proliferation capacity of hiPSCs in suspension culture gradually decreased over time, whereas cell proliferation capacity was well maintained in poly-Z culture (Figs. S8 and S9). A key benefit of our poly-Z culture system in comparison with conventional culture methods is that the hiPSC spheroids derived from poly-Z display a genetic profile resembling that of naïve pluripotent stem cells (Figs. 4C and 5). In contrast, both VN-cultured hiPSC colonies and suspension-cultured hiPSC spheroids exhibited up-regulation of gene markers associated with the primed pluripotent state (Fig. 5). Considering that the naïve state of hiPSCs possesses greater differentiation potential than their primed state [17], poly-Z-cultured hiPSCs with naïve-like features can be an attractive cell source for human development research and regenerative medicine [18–22].

Matrigel and VN are widely used for hPSC culture but have limitations such as undefined components and high costs [8,9]. Synthetic polymer-based culture systems address these issues by providing a defined environment for hPSC expansion, differentiation, and maintenance. For instance, xeno-free polyvinyl butyral-based polymers eliminate animal-derived components while supporting hPSC attachment [45], and PNIPAAm-PEG-based thermoresponsive hydrogels enable long-term 3D spheroid culture with high expansion rates [46]. However, most synthetic polymer studies focus on 2D adherent conditions, with limited investigation into the pluripotency state. Our study demonstrates that hiPSCs can be cultured as 3D spheroids on poly-Z, exhibiting genetic traits of naïve pluripotency.

Surface properties such as stiffness, topography, and hydrophobicity notably impact hPSC behavior. Stiff hydrogels promote adhesion and self-renewal [47], while nanotopography influences pluripotency maintenance [48]. Hydrophobic surfaces can facilitate suspension culture by preventing adhesion, as albumin in the medium adsorbs onto hydrophobic surfaces, rendering them superhydrophilic [49]. This aligns with our observation that poly-Z, a hydrophobic surface with a water contact angle of ~102° (Fig. S1), supports hiPSC spheroid formation, likely due to albumin adsorption. Notably, poly-Z-derived spheroids exhibited naïve-like pluripotency. While 3D environments have been suggested to support naïve pluripotency [50], our results indicate that spheroid formation alone is not sufficient. hiPSC spheroids cultured in conventional suspension medium did not show naïve-like traits, unlike those on poly-Z (Fig. 5), suggesting that the pluripotency state depends on the specific mechanism of 3D spheroid formation. Further studies are needed to elucidate these mechanisms, and poly-Z represents a promising platform for generating hiPSC spheroids for stem cell research and regenerative medicine.

We also investigated the underlying biological processes involved in the promotion of naïve-like features in hiPSC spheroids cultured on poly-Z. A GSEA revealed clear down-regulation of genes associated with focal adhesion and ECM receptor interactions (Fig. 6C to E), which are known to influence the pluripotency and self-renewal properties of hPSCs [38]. The gene expression patterns of naïve or primed pluripotency marker genes obtained following stimulation of integrin expression in hiPSCs cultured on poly-Z support the inference that down-regulated focal adhesion and ECM receptor interactions promote naïve-like pluripotency of poly-Z-cultured hiPSC spheroids (Fig. 7D and E). These findings provide valuable insights into the mechanism underlying the acquisition of naïve-like features in poly-Z-cultured hiPSC spheroids. However, it should be noted that this proof-of-concept study primarily aimed to validate the potential of a cyclosiloxane polymer matrix as a 3D culture platform for hiPSCs and to identify representative gene expression changes in hiPSC spheroids, rather than to investigate the underlying molecular mechanisms in detail. Therefore, further studies are required to elucidate the long-term effects of reduced integrin signaling on hiPSC survival, differentiation potential, and induction of naïve pluripotency on other culture substrates, particularly at the level of intracellular signaling alterations. Additionally, comprehensive analyses of the genomic stability and tumorigenicity of hiPSC spheroids, such as sub-chromosomal analysis and assessments of terminal differentiation levels and long-term tumorigenicity in vivo, will be necessary. Such investigations will contribute to a deeper understanding of the intricate intracellular mechanisms driving the acquisition of naïve-like pluripotency in hiPSC spheroids and facilitate their future clinical application.

In conclusion, our study presents a novel culture platform for hiPSCs that utilizes a cross-linked cyclosiloxane polymer matrix to promote the formation of hiPSC spheroids and facilitate the long-term maintenance of pluripotency with naïve-like features. Notably, the hiPSC spheroids cultured on this polymer matrix can be directly differentiated into all 3 germline cell types. One notable advantage of our culture platform is its scalability, cost-effectiveness, and versatility in accommodating different-shaped culture plates. The ease of preparation and the potential for large-scale production make our polymer matrix an attractive option for advancing human pluripotent stem cell research and regenerative medicine applications.

## Data Availability

Data will be made available on request. The mRNA-sequencing data are available at the National Center for Biotechnology Information Gene Expression Omnibus database (accession code GSE233565).
