# Supplementary material for: A Cross-Linked Cyclosiloxane Polymer Matrix as a Platform Enabling Long-Term Culture of Human Induced Pluripotent Stem Cells with Naïve-Like Features
Source: Biomater Res. 2025 Apr 28;29:0197. doi: 10.34133/bmr.0197 (PMC12034926; doi:10.34133/bmr.0197)
Supplement: Supplementary 1 — Figs. S1 to S10 Tables S1 to S4 [file bmr.0197.f1.docx]

**Supplementary Material**

**A Cross-Linked Cyclosiloxane Polymer Matrix as a Platform Enabling Long-Term Culture of Human Induced Pluripotent Stem Cells with Naïve-like Features**

Changjin Seo^1,2†^, Junhyuk Song^1,2†^, Yoonjung Choi^3^, Taemook Kim^3^, Daeyoup Lee^1*^ and Sangyong Jon^1,2*^

^1^ Department of Biological Sciences, KAIST Institute for the BioCentury, Korea Advanced Institute of Science and Technology (KAIST), 291 Daehak-ro, Daejeon 34141, Republic of Korea

^2^ Center for Precision Bio-Nanomedicine, Korea Advanced Institute of Science and Technology (KAIST), 291 Daehak-ro, Daejeon 34141, Republic of Korea

^3^ Deargen Inc. R1846, 18F, 136 Cheongsa-ro, Seo-gu, Daejeon 35220, Republic of Korea

*Address correspondence to: daeyoup@kaist.ac.kr (Daeyoup Lee), syjon@kaist.ac.kr (Sangyong Jon)

^†^These authors contributed equally to this work.

**Supplementary figures** S1-S10

**Supplementary tables** S1-S4

**
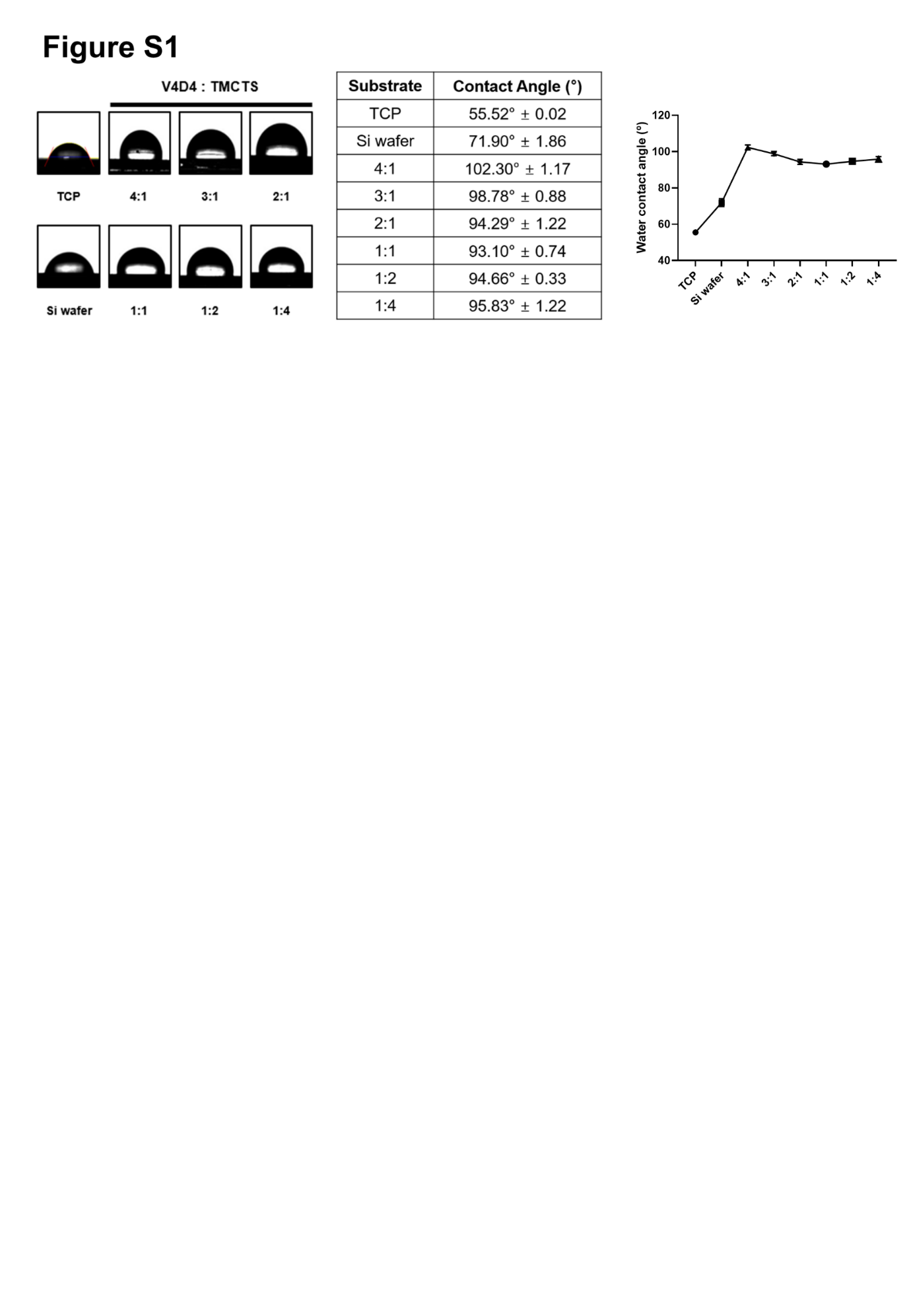
**

**Fig. S1.** Surface wettability of cross-linked cyclosiloxane polymers. Water contact angles of tissue culture plate, silicon wafer, and cross-linked cyclosiloxane polymers synthesized at various ratios of V4D4 and TMCTS. All experiments were performed in triplicate.

**
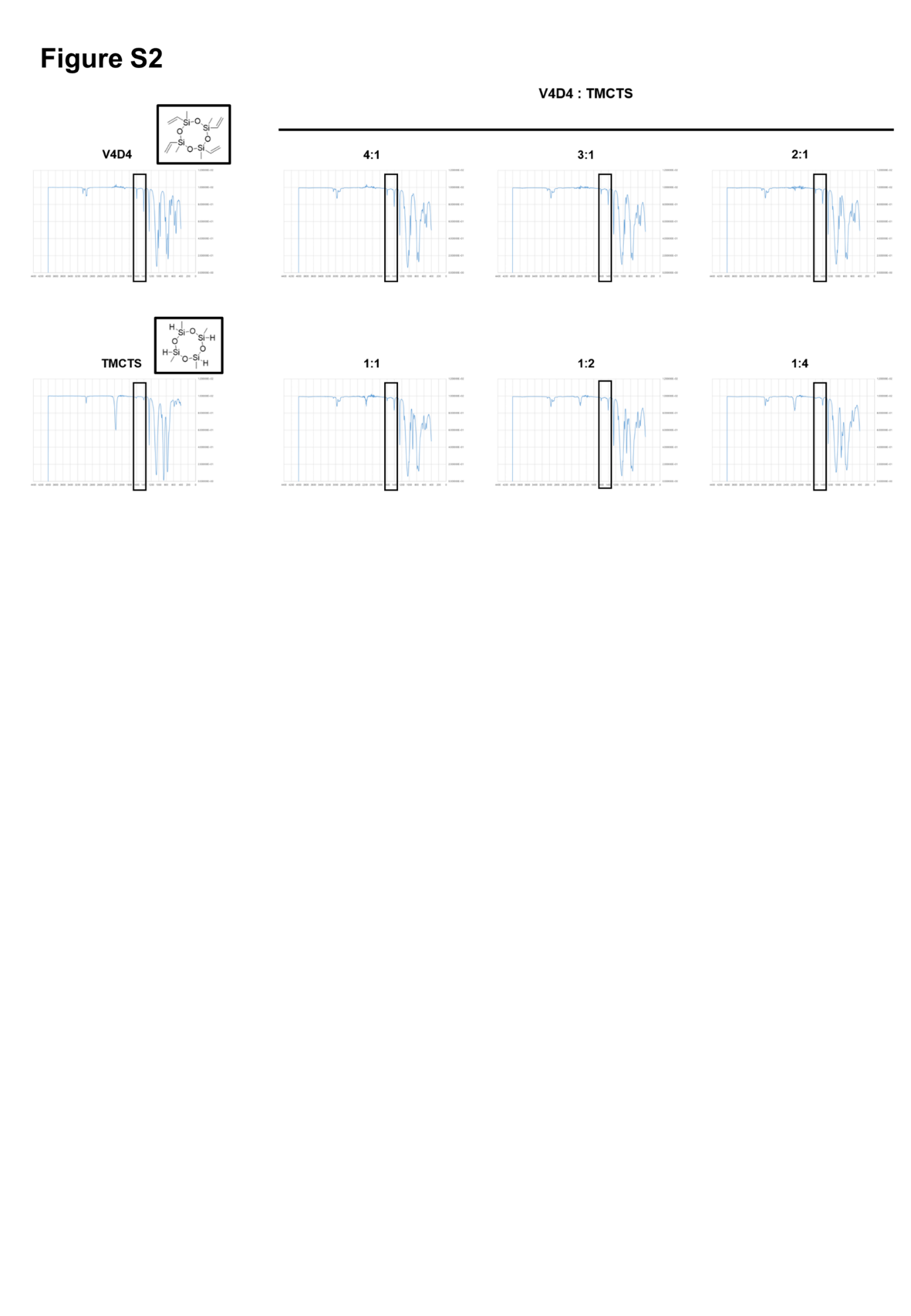
**

**Fig. S2.** FT-IR spectra of V4D4, TMCTS, and cross-linked cyclosiloxane polymers synthesized at various ratios of V4D4 and TMCTS.

**
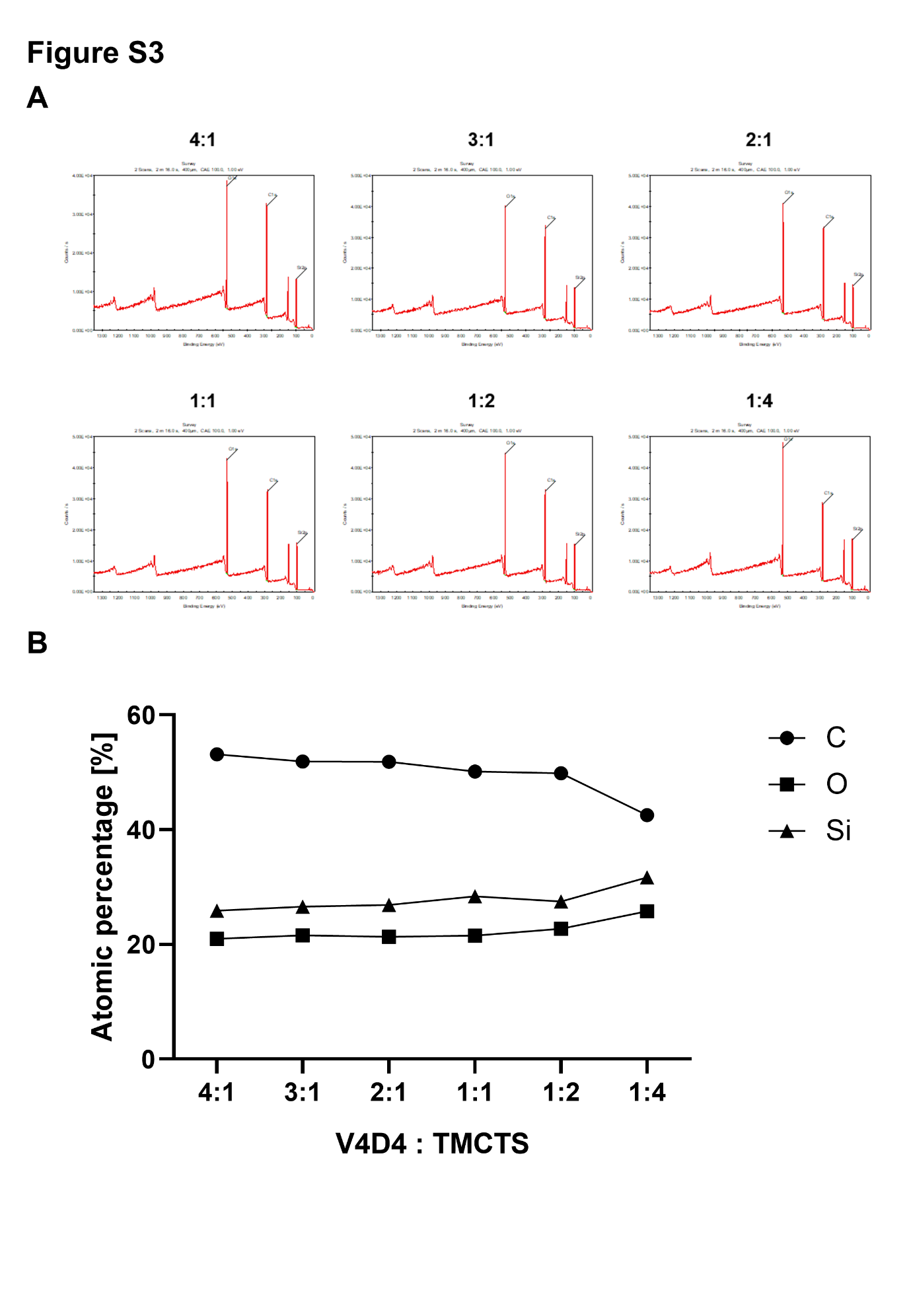
**

**Fig. S3.** XPS scan of cross-linked cyclosiloxane polymers. A) XPS spectra of cross-linked cyclosiloxane polymers synthesized at various ratios of V4D4 and TMCTS. B) Atomic composition of cross-linked cyclosiloxane polymers obtained from XPS analysis.


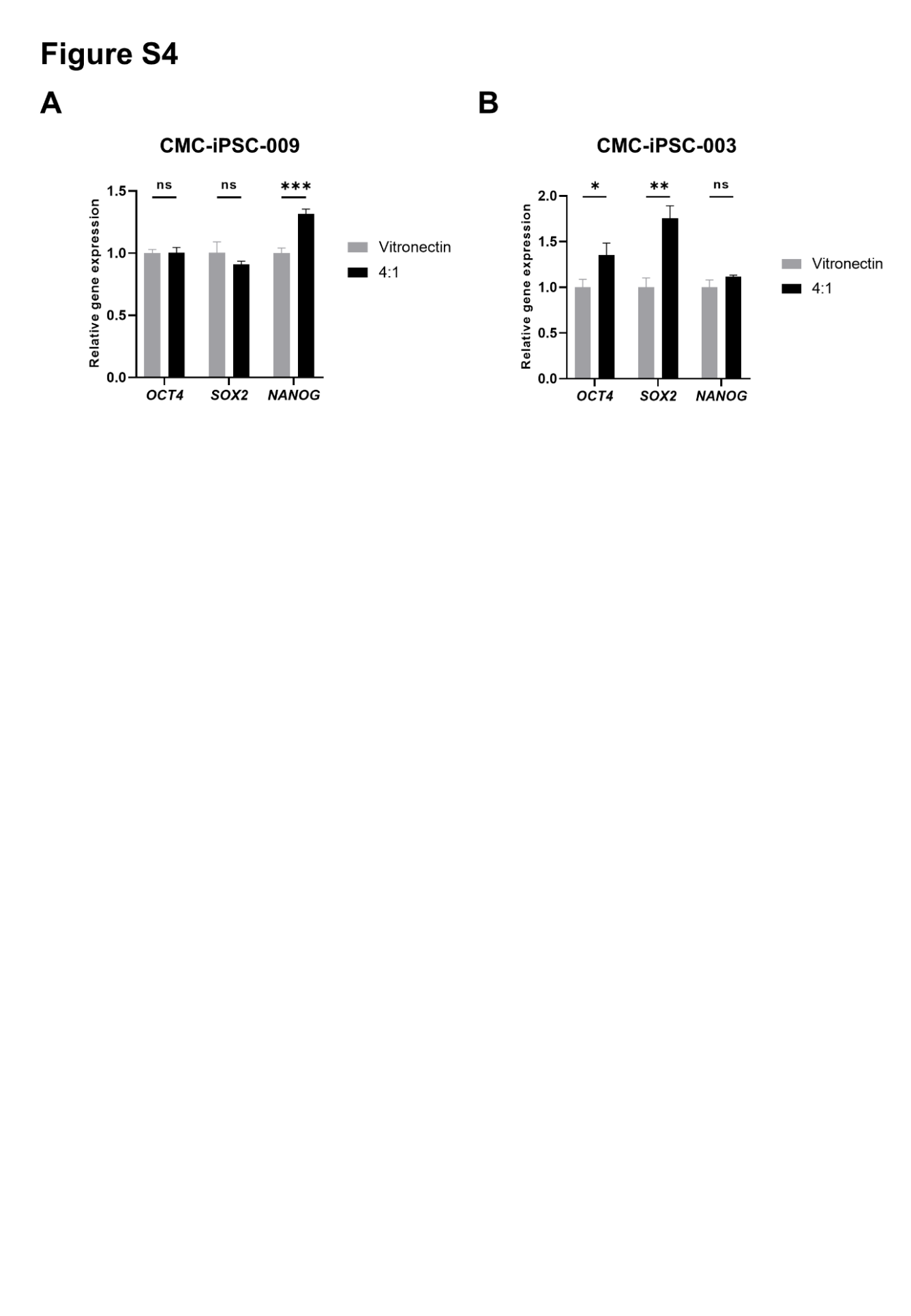


**Fig. S4.** Relative expression of pluripotency genes in hiPSCs cultured for 4 days on polymers synthesized at a V4D4/TMCTS ratio of 4:1. A) qRT-PCR results for pluripotency markers in CMC-iPSC-009 cell lines. B) qRT-PCR results for pluripotency markers in CMC-iPSC-003 cell lines. All experiments were performed in triplicate. Results in (A, B) represent the means ± SD of three independent experiments (ns: not significant; *p < 0.05, **p < 0.01, ***p < 0.001).


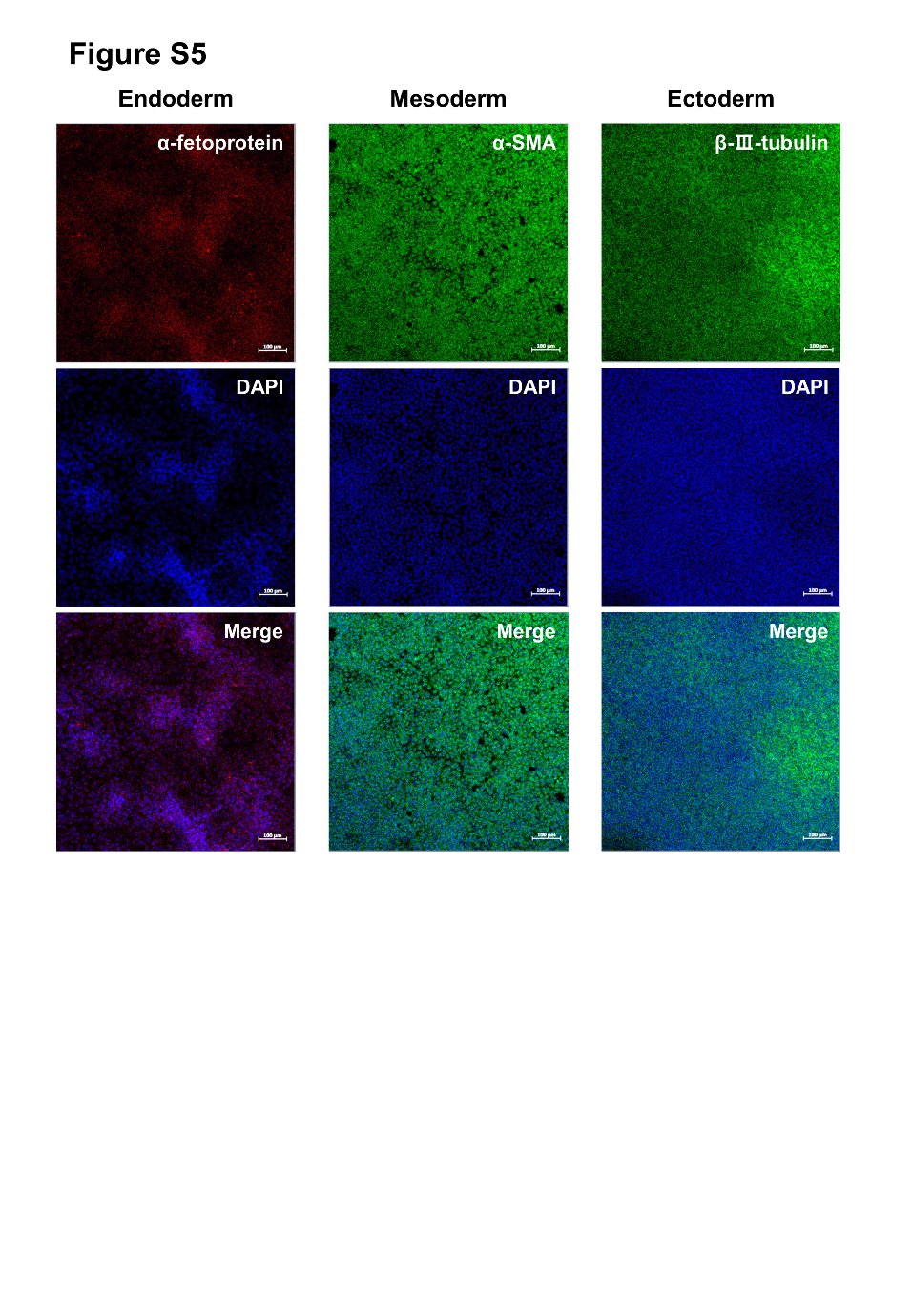


**Fig. S5.** Immunocytochemical analysis of three germ layer markers after trilineage differentiation of VN-cultured hiPSCs (CMC-iPSC-009). Scale bars: 100 µm.


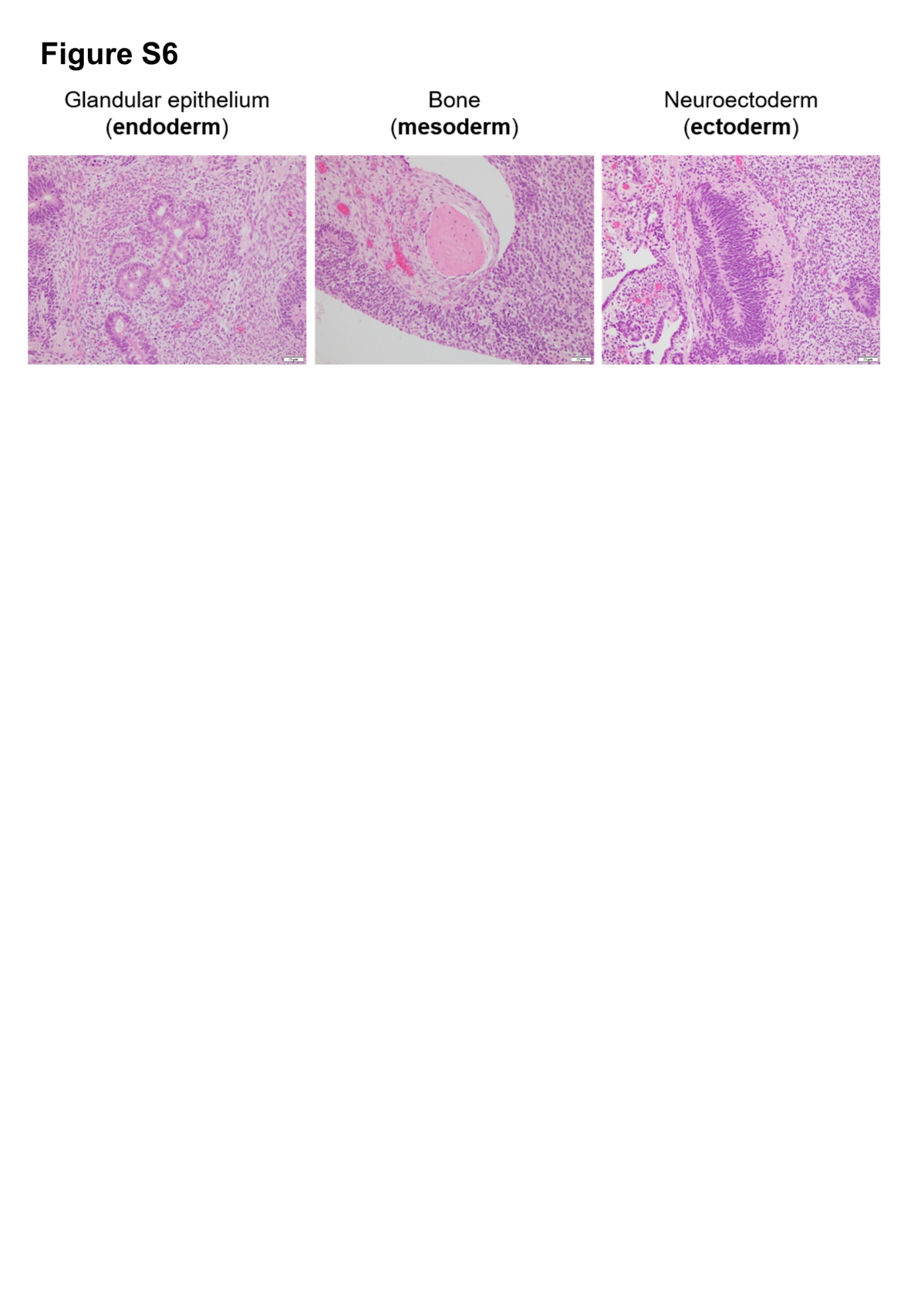


**Fig. S6.** Hematoxylin and eosin staining of teratomas derived from VN-cultured hiPSCs (CMC-iPSC-009). Scale bars: 50 µm.


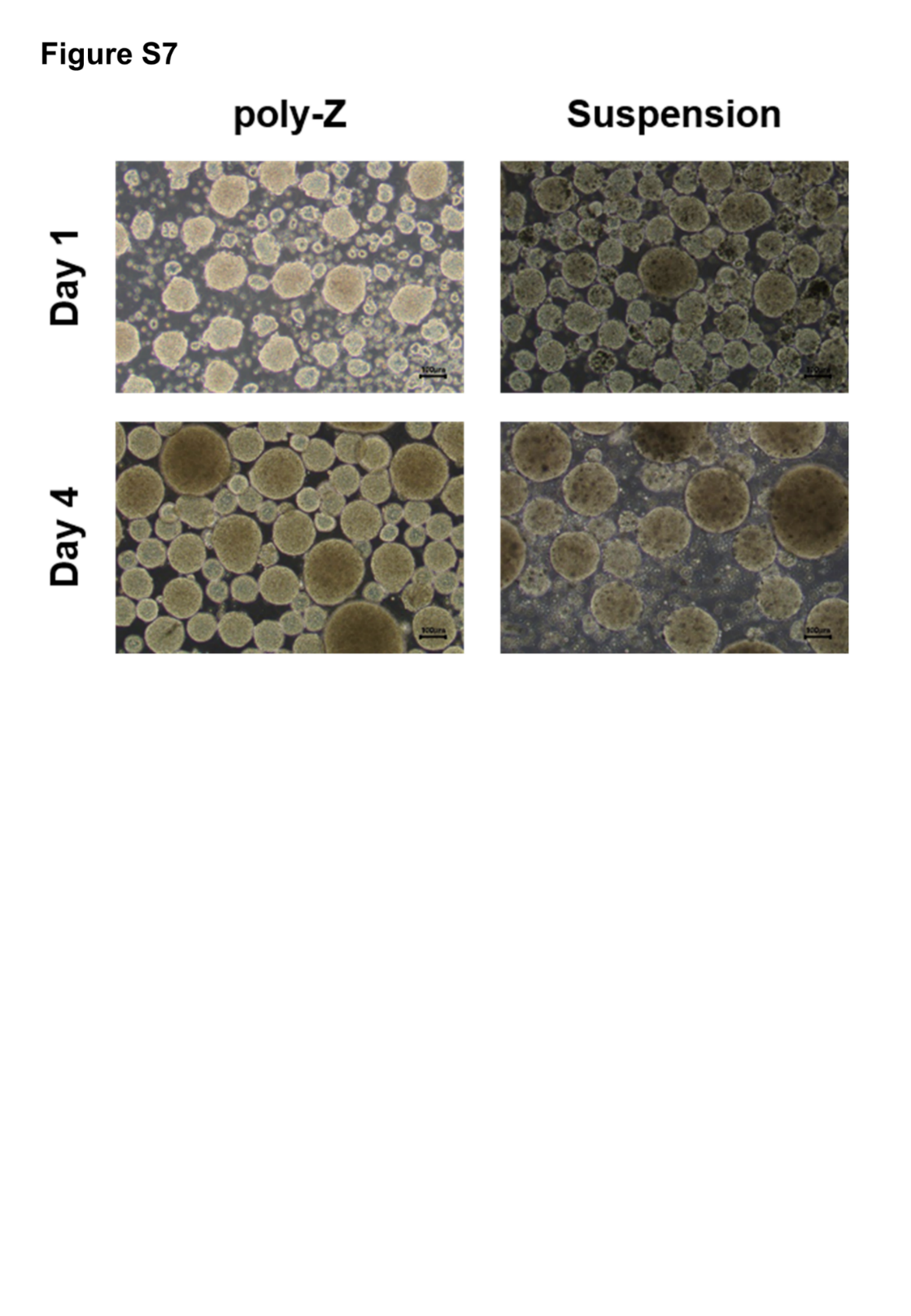


**Fig. S7.** Cell morphology of hiPSC (CMC-iPSC-009) spheroids cultured for 1 and 4 days on poly-Z or in suspension culture. Scale bar: 100µm.


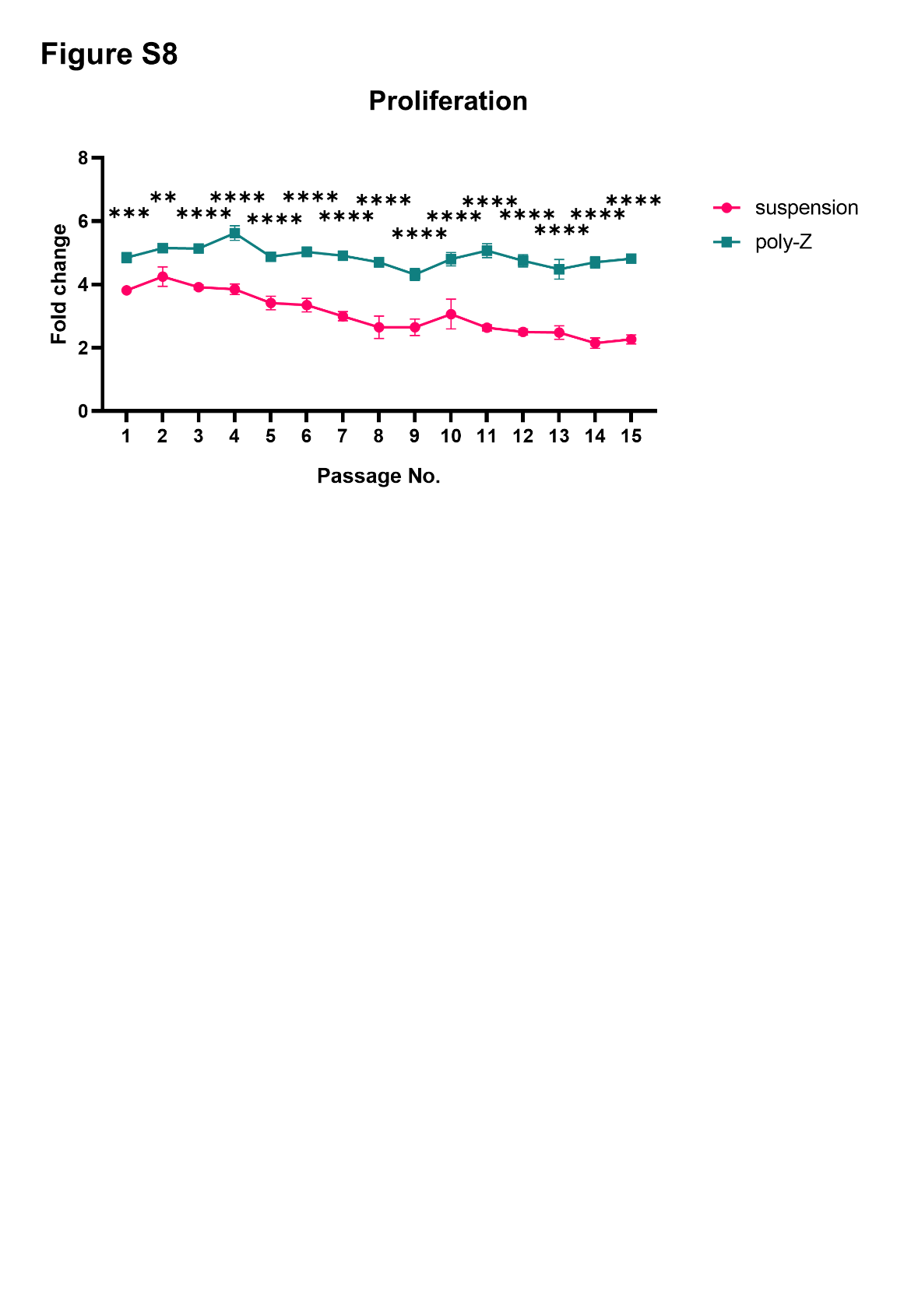


**Fig. S8.** Cell proliferation rate for each passage of hiPSC (CMC-iPSC-009) spheroids cultured on poly-Z or in suspension culture. Fold change values were calculated by dividing the total cell numbers in hiPSC spheroids on day 4 in each passage by the number of seeded cells. Cell proliferation measurements were performed in duplicate. The result presents the means ± SD of two independent experiments (**p < 0.01, ***p < 0.001, ****p < 0.0001).


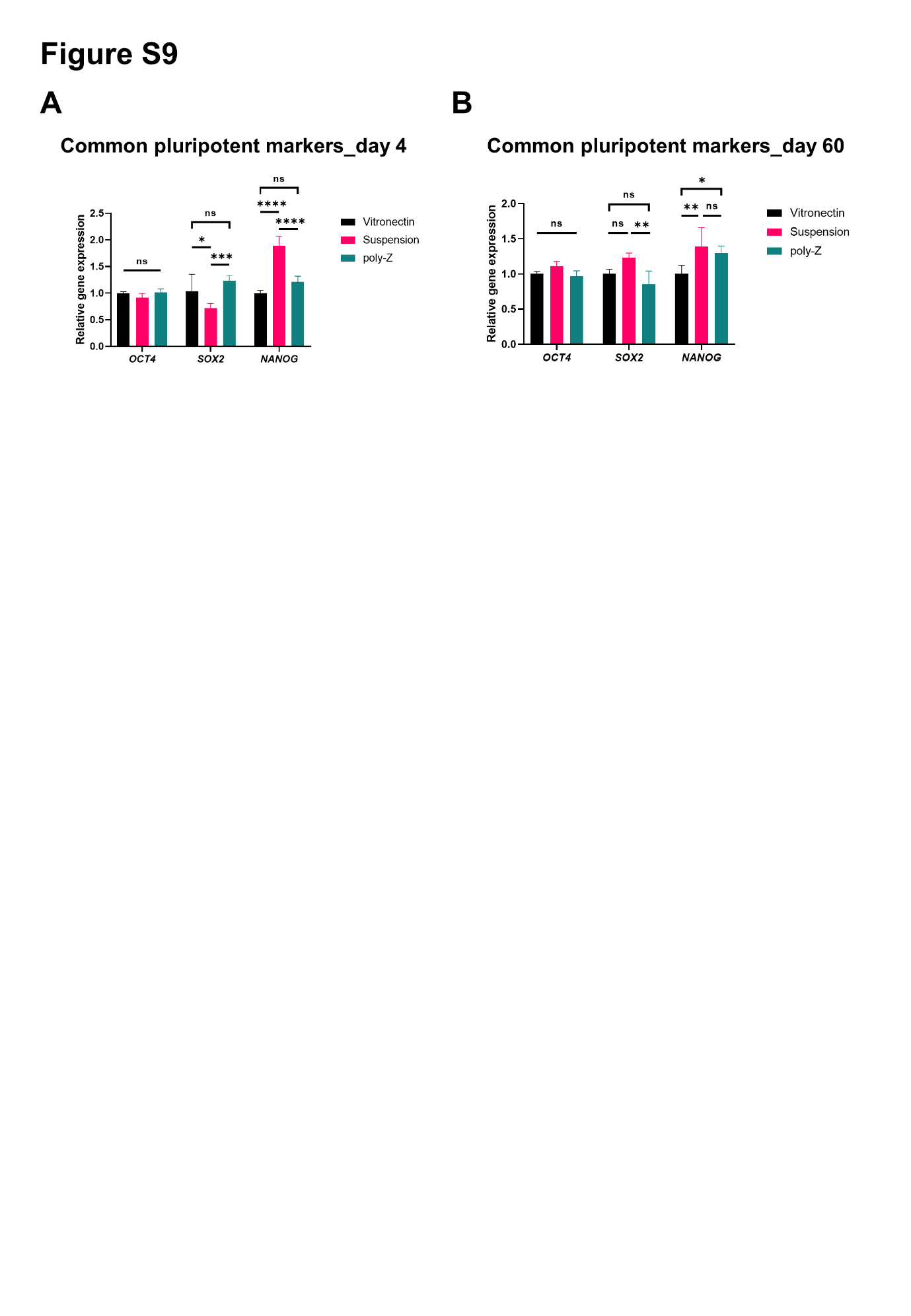


**Fig. S9.** Relative expression of pluripotency genes in hiPSCs (CMC-iPSC-009) cultured on VN, in suspension culture, or on poly-Z at different time points. A) qRT-PCR results for pluripotency genes in hiPSCs cultured on each plate type for 4 days. B) qRT-PCR results for pluripotency genes in hiPSCs cultured on each plate type for 60 days. All experiments were performed in triplicate. Results in (A, B) represents the means ± SD of three independent experiments (ns: not significant; *p < 0.05, **p < 0.01, ***p < 0.001, ****p < 0.0001).


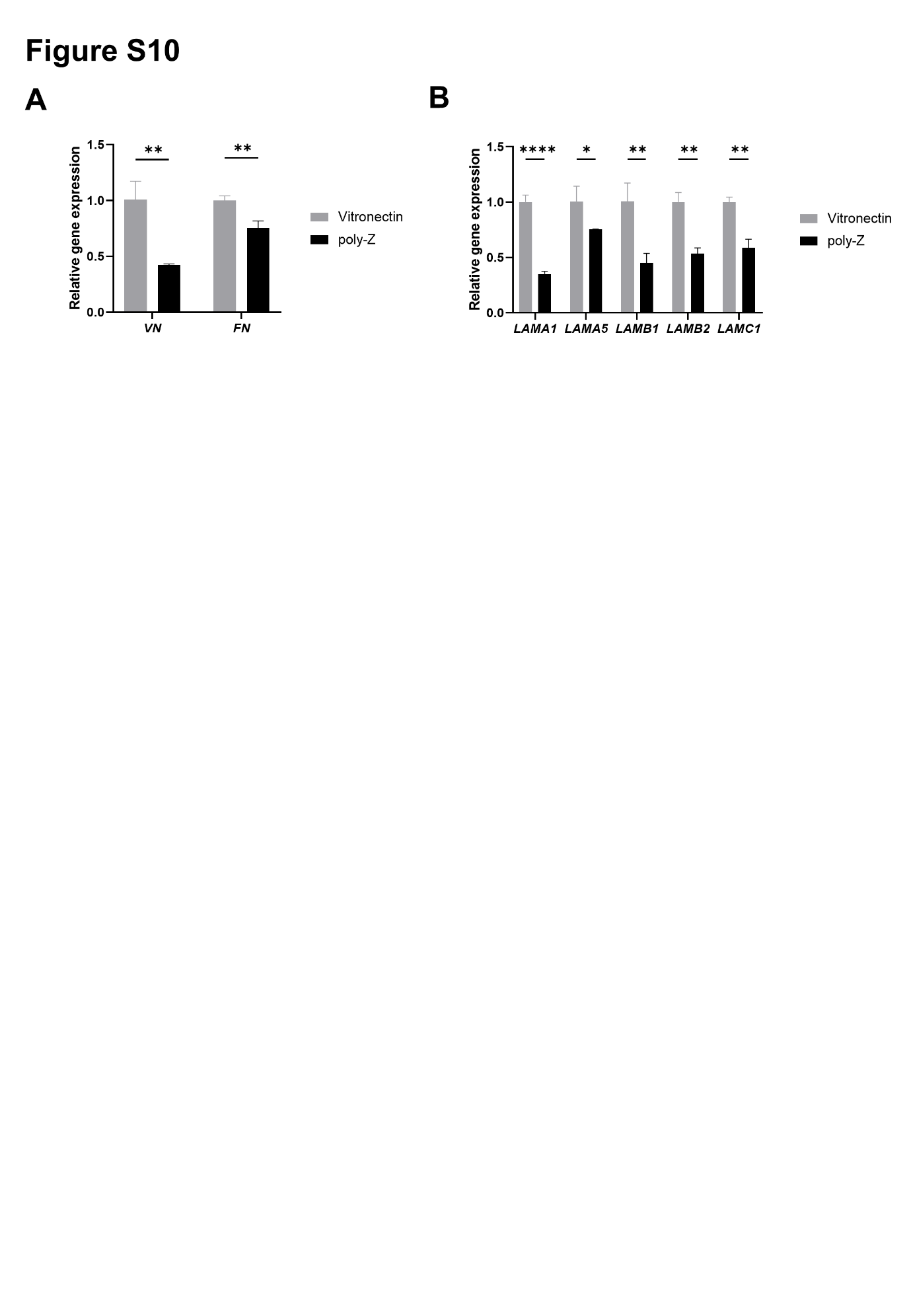


**Fig. S10.** Relative expression of ECM genes in poly-Z–cultured hiPSC (CMC-iPSC-009) spheroids. A) qRT-PCR results for vitronectin and fibronectin genes in poly-Z–cultured hiPSC spheroids. B) qRT-PCR results for laminin subunit genes in poly-Z–cultured hiPSC spheroids. All experiments were performed in triplicate. Results in (A, B) represent the means ± SD of three independent experiments (*p < 0.05, **p < 0.01, ****p < 0.0001).

**Table S1.** Primer sequences for qRT-PCR

| **Human gene** | **Primer pair** | **Primer sequences (5’ – 3’)** |
| --- | --- | --- |
| *GAPDH* | Forward primer | TGGACCTGACCTGCCGTCTA |
|  | Reverse primer | CCCTGTTGCTGTAGCCAAATTC |
| *OCT4* | Forward primer | AAGCGAACCAGTATCGAGAACC |
|  | Reverse primer | CTGATCTGCTGCAGTGTGGGT |
| *SOX2* | Forward primer | GGCAATAGCATGGCGAGC |
|  | Reverse primer | TTCATGTGCGCGTAACTGTC |
| *NANOG* | Forward primer | AATACCTCAGCCTCCAGCAGATG |
|  | Reverse primer | TGCGTCACACCATTGCTATTCTTC |
| *ITGA1* | Forward primer | CCGATCCAGAAAATGGGCCT |
|  | Reverse primer | CTGGGATCGGACAATCAGCA |
| *ITGA6* | Forward primer | AGCTGTGCTTGCTCTACCTG |
|  | Reverse primer | AACGCTGGTCAAAAACAGCA |
| *ITGAV* | Forward primer | CACCCTCCTTCTGATCCTGTA |
|  | Reverse primer | TGTCGTCTGGAAGTCTCCTG |
| *ITGB1* | Forward primer | ACTGCAAGAACGGGGTGAAT |
|  | Reverse primer | CAGACACCACACTCGCAGAT |
| *ITGB5* | Forward primer | AGCGTGTACCAGAACCTGTG |
|  | Reverse primer | GCAGTTACAGTTGTCCCCGA |
| *CDH1* | Forward primer | AGCACGTACACAGCCCTAA |
|  | Reverse primer | GGTATGGGGGCGTTGTCA |
| *β-actin* | Forward primer | GGCTGTGCTATCCCTGTACG |
|  | Reverse primer | CTTGATCTTCATTGTGCTGGGTG |
| *DPPA3* | Forward primer | TGTTACTCGGCGGAGTTCGTA |
|  | Reverse primer | CCATCCATTAGACACGCAGAAA |
| *DPPA5* | Forward primer | GTCGTGGTTTACGGCTCCTAT |
|  | Reverse primer | GGCAAGTTTGAGCATCCCTC |
| *ZIC2* | Forward primer | CCGGAGTCTTTGAAGCTGAA |
|  | Reverse primer | AACGTGGGCATGGAGATTAG |
| *SOX11* | Forward primer | CTCAAGCACATGGCCGACTA |
|  | Reverse primer | GCACTTTGGCGACGTTGTAG |
| *VTN* | Forward primer | TATACGGCTGAGTGCAAGCC |
|  | Reverse primer | TTTTCTCCTCGCCATCGTCA |
| *FN1* | Forward primer | GCCACCCCCATAAGGCATAG |
|  | Reverse primer | CCGTGTGGGTACAGGTGATAG |
| *LAMA1* | Forward primer | ATATGAGCCCAAAACCGCCA |
|  | Reverse primer | GCGTTCCCGTCAACAATCAG |
| *LAMA5* | Forward primer | TGCCACCGACTGTTACTACG |
|  | Reverse primer | GTGCTGGCAGTCGATACAGA |
| *LAMB1* | Forward primer | GCTTTCAGTTTCTTAGCCCTGTG |
|  | Reverse primer | ATGAGAAGGTCGCCCGTG |
| *LAMB2* | Forward primer | CCCCTACAGCTCACGGATTC |
|  | Reverse primer | CGTGGGTCGAGTAGGTTGTC |
| *LAMC1* | Forward primer | ACCTACTCCAAGGCAAACCG |
|  | Reverse primer | CCCCAGTGAGGGGAGAAATG |

**Table S2.** List of antibodies

| **Antibody** | **Manufacturer**  **(Catalogue No.)** | **Application** | **Dilution** |
| --- | --- | --- | --- |
| **Primary antibodies** |  |  |  |
| Anti-OCT4 (host: rabbit) | Invitrogen  (A24867) | ICC | 1/200 |
| Anti-SOX2 (host: rat) | Invitrogen  (A24759) | ICC | 1/100 |
| Anti-TRA-1-60 (host: mouse IgM) | Invitrogen  (A24868) | ICC | 1/100 |
| Anti-SSEA-4 (host: mouse IgG3) | Invitrogen  (A24866) | ICC | 1/100 |
| Anti-SSEA-4 (host: mouse IgG3) | R&D Systems  (MAB1435) | FC | 1/400 |
| Anti-α-fetoprotein (host: mouse IgG1) | Abcam  (ab3980) | ICC | 1/200 |
| Anti-α-smooth muscle actin (host: rabbit) | Abcam  (ab5694) | ICC | 1/100 |
| Anti-β-III-tubulin (host: rabbit) | Abcam  (ab18207) | ICC | 1/200 |
| Anti-GAPDH (host: rabbit) | Cell Signaling Technology  (2118S) | WB | 1/1000 |
| Anti-Stella(DPPA3) (host: rabbit) | Invitrogen  (PA5-34601) | WB | 1/1000 |
| Anti-DPPA5 (host: mouse IgG1) | R&D Systems  (MAB3125) | WB | 1/1000 |
| Anti-OCT4 (host: rabbit) | Abcam  (ab200834) | WB | 1/10000 |
| Anti-TRA-1-60, PE | Stemcell Technologies  (ST60064PE) | FC | 1/20 |
| Anti-TRA-1-81, Alexa Fluor 488 | Stemcell Technologies  (ST60065AD) | FC | 1/20 |
| **Secondary antibodies** |  |  |  |
| Alexa Fluor 594 donkey anti-rabbit | Invitrogen  (A24870) | ICC | 1/250 |
| Alexa Fluor 488 goat anti-mouse IgG3 | Invitrogen  (A24877) | ICC | 1/250 |
| Alexa Fluor 488 donkey anti-rat | Invitrogen  (A24876) | ICC | 1/250 |
| Alexa Fluor 594 goat anti-mouse IgM | Invitrogen  (A24872) | ICC | 1/250 |
| Alexa Fluor 488 goat anti-rabbit | Abcam  (ab150081) | ICC | 1/500 |
| Alexa Fluor 594 goat anti-mouse IgG | Abcam  (ab150116) | ICC, FC | 1/500, 1/4000 |
| Goat Anti-Rabbit IgG H&L (HRP) | Abcam  (ab205718) | WB | 1/20000 |
| Goat Anti-Mouse IgG H&L (HRP) | Invitrogen  (31430) | WB | 1/20000 |

ICC, immunocytochemistry; FC, flow cytometry; WB, western blot

**Table S3.** Atomic composition of cross-linked cyclosiloxane polymers calculated from the XPS analysis.

| **V4D4 : TMCTS** | **Atoms** | **Measured value (%)** |
| --- | --- | --- |
| 4:1 | C | 53.14 |
|  | O | 20.97 |
|  | Si | 25.9 |
| 3:1 | C | 51.88 |
|  | O | 21.57 |
|  | Si | 26.55 |
| 2:1 | C | 51.83 |
|  | O | 21.3 |
|  | Si | 26.88 |
| 1:1 | C | 50.12 |
|  | O | 21.53 |
|  | Si | 28.35 |
| 1:2 | C | 49.82 |
|  | O | 22.73 |
|  | Si | 27.46 |
| 1:4 | C | 42.55 |
|  | O | 25.76 |
|  | Si | 31.69 |

**Table S4.** Total cell numbers at each passage for hiPSCs (CMC-iPSC-009) cultured on poly-Z or in suspension culture for 60 days

|  | **poly-Z** | **Suspension** |
| --- | --- | --- |
| Seeding number | 8.00 × 10^5^ cells | 3.00 × 10^5^ cells |
| Passage 1  (day 4) | 3.88 × 10^6^ cells | 1.15 × 10^6^ cells |
| Passage 2  (day 8) | 4.12 × 10^6^ cells | 1.28 × 10^6^ cells |
| Passage 3  (day 12) | 4.11 × 10^6^ cells | 1.18 × 10^6^ cells |
| Passage 4  (day 16) | 4.50 × 10^6^ cells | 1.16 × 10^6^ cells |
| Passage 5  (day 20) | 3.90 × 10^6^ cells | 1.03 × 10^6^ cells |
| Passage 6  (day 24) | 4.03 × 10^6^ cells | 1.01 × 10^6^ cells |
| Passage 7  (day 28) | 3.93 × 10^6^ cells | 9.00 × 10^5^ cells |
| Passage 8  (day 32) | 3.77 × 10^6^ cells | 7.95 × 10^5^ cells |
| Passage 9  (day 36) | 3.46 × 10^6^ cells | 7.95 × 10^5^ cells |
| Passage 10  (day 40) | 3.84 × 10^6^ cells | 9.20 × 10^5^ cells |
| Passage 11  (day 44) | 4.06 × 10^6^ cells | 7.90 × 10^5^ cells |
| Passage 12  (day 48) | 3.80 × 10^6^ cells | 7.50 × 10^5^ cells |
| Passage 13  (day 52) | 3.59 × 10^6^ cells | 7.45 × 10^5^ cells |
| Passage 14  (day 56) | 3.76 × 10^6^ cells | 6.45 × 10^5^ cells |
| Passage 15  (day 60) | 3.86 × 10^6^ cells | 6.80 × 10^5^ cells |
